# Impact of additives on syntrophic propionate and acetate enrichments under high-ammonia conditions

**DOI:** 10.1007/s00253-024-13263-7

**Published:** 2024-08-07

**Authors:** Eduardo Pinela, Anna Schnürer, Anna Neubeck, Jan Moestedt, Maria Westerholm

**Affiliations:** 1 Department of Molecular Sciences, Swedish University of Agricultural Sciences, 750 07 Uppsala, Sweden; 2 Department of Earth Sciences, Uppsala University, 752 36 Uppsala, Sweden; 3 Department of Biogas R & D, Tekniska Verken I Linköping AB (Publ.), Box 1500, 581 15 Linköping, Sweden; 4 Department of Thematic Studies - Environmental Change, Linköping University, 581 83 Linköping, Sweden

**Keywords:** Syntrophy, Zeolite, Graphene, Iron oxide, Biogas

## Abstract

**Abstract:**

High ammonia concentrations in anaerobic degradation systems cause volatile fatty acid accumulation and reduced methane yield, which often derive from restricted activity of syntrophic acid-oxidising bacteria and hydrogenotrophic methanogens. Inclusion of additives that facilitate the electron transfer or increase cell proximity of syntrophic species by flocculation can be a suitable strategy to counteract these problems, but its actual impact on syntrophic interactions has yet to be determined. In this study, microbial cultivation and molecular and microscopic analysis were performed to evaluate the impact of conductive (graphene, iron oxide) and non-conductive (zeolite) additives on the degradation rate of acetate and propionate to methane by highly enriched ammonia-tolerant syntrophic cultures derived from a biogas process. All additives had a low impact on the lag phase but resulted in a higher rate of acetate (except graphene) and propionate degradation. The syntrophic bacteria ‘*Candidatus* Syntrophopropionicum ammoniitolerans’, *Syntrophaceticus schinkii* and a novel hydrogenotrophic methanogen were found in higher relative abundance and higher gene copy numbers in flocculating communities than in planktonic communities in the cultures, indicating benefits to syntrophs of living in close proximity to their cooperating partner. Microscopy and element analysis showed precipitation of phosphates and biofilm formation in all batches except on the graphene batches, possibly enhancing the rate of acetate and propionate degradation. Overall, the concordance of responses observed in both acetate- and propionate-fed cultures highlight the suitability of the addition of iron oxide or zeolites to enhance acid conversion to methane in high-ammonia biogas processes.

**Key points:**

•* All additives promoted acetate (except graphene) and propionate degradation.*

•* A preference for floc formation by ammonia-tolerant syntrophs was revealed.*

•* Microbes colonised the surfaces of iron oxide and zeolite, but not graphene.*

**Graphical Abstract:**

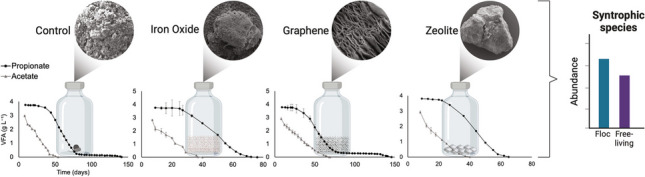

**Supplementary Information:**

The online version contains supplementary material available at 10.1007/s00253-024-13263-7.

## Introduction

Nitrogen-rich wastes, such as slaughterhouse waste, animal manure and food waste, are promising substrates for biogas production due to their high methane potential and associated generation of nutrient-rich, high-qualitative fertiliser (Garcia et al. 2019). However, breakdown of nitrogenous compounds in the biogas process gives rise to ammonia (NH_3_), a compound that is important for microbial growth but inhibitory at elevated concentrations for some microbial groups (Yenigün and Demirel 2013). In solution, ammonia and its ionised form, ammonium (NH_4_^+^), exist in equilibrium that is dependent on temperature and pH, with higher temperatures and higher pH shifting the equilibrium towards ammonia, the most toxic of the two compounds for anaerobic microorganisms (Kayhanian 1999). Elevated ammonia levels can affect many microbial groups responsible for the early stages of anaerobic digestion, such as hydrolysis and acidogenesis. In later stages, volatile fatty acids (VFA) such as propionate and acetate are converted into smaller compounds such as acetate (in the case of propionate degradation), formate, H_2_ and CO_2_. The negative impact of ammonia on process performance and biogas yield derives mainly from its impact on methanogens, which are responsible for the last step in biogas production, with aceticlastic methanogens, which utilise acetate to produce CO_2_ and methane, being particularly sensitive (Yenigün and Demirel 2013). Ammonia inhibition can thus lead to accumulation of VFAs, decreased methane yield and reduced overall process efficiency (Rajagopal et al. 2013; Jiang et al. 2019). Under high-ammonia conditions, aceticlastic methanogens are outcompeted by ammonia-tolerant microorganisms, such as syntrophic acetate-oxidising bacteria (SAOB), which convert acetate to CO_2_ and formate/H_2_ (Westerholm et al. 2011a, b; Wang et al. 2015). However, these microorganisms cannot perform acetate oxidation if formate/H_2_ levels are too high and therefore require a close association with hydrogenotrophic methanogens, which consume and maintain formate/H_2_ at low levels, preventing acetate accumulation (Westerholm et al. 2015; Bonk et al. 2018; Singh et al. 2021). Another VFA that easily accumulates under high ammonia concentration is propionate, which is converted by syntrophic propionate-oxidising bacteria (SPOB) both under high- and low-ammonia conditions (albeit by different species) into CO_2_, formate/H_2_ and acetate. Similarly to acetate oxidation by SAOB, propionate oxidation by SPOB requires a close association with hydrogenotrophic methanogens to avoid thermodynamic constraints (Dolfing 1992; Si et al. 2005; Westerholm et al. 2022). The reason for propionate accumulation under high ammonia concentrations remains unclear, but it may be caused by direct ammonia inhibition of certain SPOB (Bonk et al. 2018; Zhang et al. 2018). However, even when an ammonia-tolerant SPOB is present, propionate degradation can be hampered due to ammonia-related stress on SPOB or indirectly through reduced methanogenic activity (Liu et al. 1999; Westerholm et al. 2022), resulting in build-up of acetate and propionate and loss of methane potential in high-ammonia processes.

It has been suggested that the VFA degradation rate in anaerobic processes can be improved by facilitating and increasing exchange of the electron-carrying intermediary compounds formate (Thiele and Zeikus 1988) and H_2_ (Dolfing 2013), by reducing cell-to-cell distances between syntrophic microorganisms. Another mechanism for electron allocation between different microbial species is the direct interspecies electron transfer (DIET) through e-pili (Reguera et al. 2005) or outer-membrane transport proteins (Nevin and Lovley 2000). Even though DIET has been proposed to provide a slight thermodynamic advantage compared to using intermediary electron-carrying compounds (Storck et al. 2016), it has been suggested that DIET and interspecies formate/H_2_ transfer can occur simultaneously (Jing et al. 2017). This would theoretically benefit syntrophic activity as it offers different strategies to transfer electrons (Viggi et al. 2014). Several SPOB and SAOB have been shown to use both formate and H_2_ for electron transfer (Hidalgo-Ahumada et al. 2018; Singh et al. 2023; Weng et al. 2024) but only *Pelotomaculum thermopropionicum* has been suggested to perform DIET, by means of conductive nanowires (e-pili) (Gorby et al. 2006), and no SAOB has yet been proven to have this capability. Similar to the use of intermediary compounds, also DIET would theoretically be promoted by cell proximity and syntrophic communities have been found aggregated in different ways (Thiele et al. 1988; Ishii et al. 2005; Weng et al. 2024), which could be a strategy of the involved microorganisms to reduce distances. Reduction in cell distance has also been proposed to be facilitated by presence of additives like zeolites that function as a platform to which the microorganisms can adhere and establish direct and indirect connections (Montalvo et al. 2012). Furthermore, some zeolites have the additional benefit of being capable of exchanging ions with toxic compounds (such as ammonia) present in the media (Wang and Peng 2010). It has also been suggested that the electrical conductivity in some additives (like graphene and iron oxide) can serve as an electron-shuttling mechanism and thereby facilitate DIET between cells (Rotaru et al. 2014; Zhuang et al. 2018). Even though the inclusion of additives has been shown to enhance the overall anaerobic digestion process (Wang et al. 2018a, b; Li et al. 2019), few studies have explicitly demonstrated that this impact derives from promotion of syntrophic acid-degrading microorganisms. This is because, in biogas processes, determining acid formation and degradation rates is complicated by the constant formation of VFAs resulting from breakdown of compounds higher up in the degradation chain. Furthermore, despite having central roles in biogas processes, syntrophs typically constitute only a few percent of the total microbial community, making them difficult to study (Westerholm et al. 2016).

The aim of the present work was to specifically examine how the presence of additives impact on ammonia-tolerant syntrophic microorganisms, with regard to their acid-degrading capacity and floc formation. This was conducted using highly enriched microbial communities of acid-oxidising bacteria (SAOB and SPOB) and hydrogenotrophic methanogens, originating from a high-ammonia biogas digester. Cultivations were conducted in batch assays under high (0.3 M) ammonia and mesophilic (37° C) conditions, with either the conductive additives graphene powder or iron oxide (II, III) nanoparticles, or the non-conductive zeolite clinoptilolite. Acetate and propionate degradation and methane formation were analysed and the impact of the additives on microbial community composition was determined for flocculating and planktonic microbial communities. Microbial samples were analysed after the batch cultivations using scanning electron microscopy (SEM) and element analysis, to further reveal the disposition of cells attached to the additives and to assess the formation of precipitates.

## Material and methods

### Source of syntrophic communities and anaerobic batch set-up

Enriched syntrophic acetate-oxidising (SAO) and syntrophic propionate-oxidising (SPO) communities used as inoculum cultures were obtained from laboratory-scale, continuously stirred tank reactors operating under anaerobic and mesophilic conditions (37 °C) as described in Singh et al. (2021). In short, the reactors were inoculated with sludge from a high-ammonia biogas process (5.4 g NH_4_^+^-N L^−1^, 0.6–0.9 g NH_3_ L^−1^) and continuously fed with bicarbonate-buffered basal medium (BM) containing 0.2 g L^−1^ yeast extract, vitamins and trace elements, two reductants to remove traces of oxygen, cysteine-HCl (0.5 g L^−1^) and Na_2_S (0.24 g L^−1^) (Westerholm et al. 2010), 0.1 M sodium acetate (SAO enrichment cultures) or sodium propionate (SPO enrichment cultures) and 0.3 M ammonium chloride (16 g NH_4_Cl L^−1^). At the end of the process, the microbial cultures were transferred from the reactors to anaerobic batch flasks while flushing with N_2_.

A cultivation experiment was conducted in 1-L anaerobic batch flasks containing 0.45 L of the aforementioned BM in anoxic conditions but with 0.3 M ammonium chloride (pH 7.3, 4.7 g NH_4_^+^-N L^−1^, 0.1–0.2 g NH_3_ L^−1^) and 50 mM sodium acetate (4.1 g L^−1^) or 50 mM sodium propionate (4.8 g L^−1^). In the iron oxide nanoparticle batches, 25 mg L^−1^ of iron oxide (Fe(II,III)_3_O_4_) nanoparticles (Sigma-Aldrich) was added; in the graphene batches, 25 mg L^−1^ of graphene nanoparticles (Sigma-Aldrich) was added; and in the zeolite batches, 5 g L^−1^ of zeolites (clinoptilolite; Zeo-Concept Ece AB) was added, under continuous N_2_ flux. After the inclusion of these additives, all flasks were closed with a rubber stop and an aluminum ring, and the gas phase was swapped to a gas mixture of CO_2_ (19.9%) and N_2_ (80.1%) at an overpressure of 0.2 atm. After autoclavation at 121 °C, for 20 min, the flasks were allowed to cool down to room temperature and 25 mL of each of the two abovementioned reductants cysteine-HCl (0.5 g L^−1^) and Na_2_S (0.24 g L^−1^) was syringe-filtered to all batches. All sets of batches were established in triplicate and triplicate control batches (without additives) were included. The batches were inoculated with 5% (v/v) of the enrichment cultures described above. The inoculum cultures were thoroughly shaken prior to inoculation to ensure a heterogeneous content and homogeneous number of cells. The set of batches with acetate as substrate was inoculated with the SAO enrichment cultures while the set of batches with propionate as substrate was inoculated with the SPO enrichment cultures. All batches were then incubated in a dark room at 37 °C without shaking. After a first period of complete acid degradation (referred to as A1 for acetate degradation and P1 for propionate degradation), all batches were starved for at least 14 days to ensure absence of acid-degrading activity, and once again fed with the respective acid in a sterile stock solution to reach 50 mM in the culture media. The batches were then again incubated at 37 °C and a second complete acid degradation (referred to as A2 for acetate degradation and P2 for propionate degradation) was performed.

In order to investigate the impact of acetate on propionate degradation, a complementary batch trial was performed without inclusion of additives. Batch flasks with medium containing 0.3 M NH_4_Cl and 95 or 47 mM propionate were prepared as described above. At both propionate levels, 0 (control), 14, 29 or 61 mM acetate were added before inoculation with 5% (v/v) of the SPO enrichment culture. All batch sets were prepared in triplicate (see Table S1 in Supplementary Information (SI)) and incubated at 37 °C without shaking.

### Analytical methods

Propionate and acetate levels were analysed using high-performance liquid chromatography (HPLC) (threshold for acid detection was 0.2 g L^−1^) and methane and CO_2_ contents were assessed through gas chromatography (GC) as described by Westerholm et al. (2010). During A1 and P1, H_2_ partial pressure measurements (*p*H_2_) were made using PP1 (Peak Performer 1, reduced gas analyser) by direct injection of 1 mL of gas sample withdrawn from the headspace of the batch flasks. Standard curves using different *p*H_2_ were prepared prior to injection of samples. During A1 and P1, HPLC and GC analyses, and H_2_ and pressure readings were performed weekly and pH measurements every 2 weeks. During A2 and P2 and in the trials investigating the impact of acetate on propionate degradation, pressure readings and HPLC and GC analyses were performed weekly.

### Acid degradation and carbon balance calculations and statistical significance

**Fig. 1:**
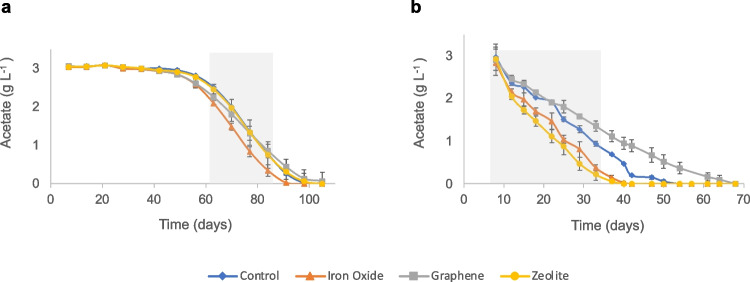
Degradation curves for the batches without (control) and with additives (iron oxide, graphene, zeolite) during **a** the first (A1) and **b** the second (A2) round of acetate degradation. Shaded areas indicate location of data points used for degradation rate calculations

**Fig. 2:**
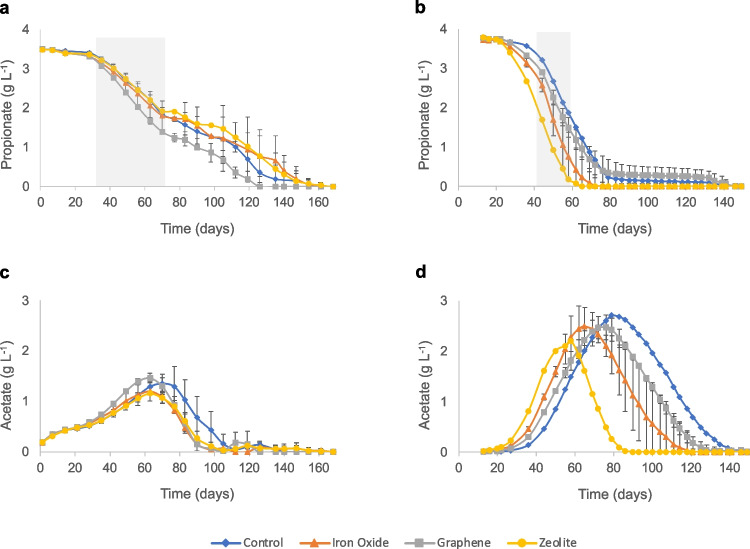
Propionate and acetate degradation curves during **a**, **c** the first (P1) and **b**, **d** the second (P2) round of propionate degradation. Shaded areas indicate location of data points used for calculation of degradation rate. Mean of triplicates ± standard deviation for all except the control and zeolite batches in **b** and **d**, which are mean of duplicates

Acid degradation rates were calculated by including the linear degradation phase (highlighted in Fig. 1a and b and Fig. 2a and b) using the equation:

1 $$\mathrm{Rate}\;\mathrm{of}\;\mathrm{acid}\;\mathrm{degradation}\;\left(\mathrm g\;\mathrm L^{-1}\mathrm{day}^{-1}\right)=\frac{(\mathrm{Ci}\;\left(\mathrm g\;\mathrm L^{-1}\right)-\mathrm{Cx}\;\left(\mathrm g\;\mathrm L^{-1}\right))}{\mathrm\Delta\;\mathrm{days}}$$

where Ci is concentration at the start of the degradation phase and Cx is concentration at a defined time point of the linear degradation phase (see Table S2 in SI for details). Carbon balance calculations were made based on the oxidation reactions of acetate or propionate to methane, which gave a theoretical conversion coefficient of 1 (Ferry 1992) and 1.75 (Singh et al. 2023), respectively (see Table S3 in SI for details). Statistical significance was assessed by single-factor ANOVA, with significance threshold set at *p* < 0.05.

### Sampling, DNA extraction and 16S rRNA gene sequencing

To avoid disturbing the syntrophic communities and acid degradation during the experiments, samples from the experimental batches were only taken at the end of A2 and P2. Microbes residing in flocculating communities were sampled by inverting the bottles to allow flocculating communities to sink to the inner side of the rubber stopper of the anaerobic flask, from which samples were withdrawn. Samples of planktonic cells were taken from the upper part of the liquid medium by gently tilting the flask halfway, leaving the flocs on the bottom of the flask. Total genomic DNA was extracted using the DNeasy Blood and Tissue Kit (Qiagen) and 16S rRNA gene sequencing was performed as described by Müller et al. (2016) using the primers 515F (5′-GTGBCAGCMGCCGCGGTAA-3′) and 805R (5′-GGACTACHVGGGTWTCTAAT-3′) (Hugerth et al. 2014). Paired-end sequencing was performed on an Illumina MiSeq instrument (Eurofins GATC Biotech GmbH, Germany) at SciLifeLab Stockholm, Sweden. Cutadapt v 4.2 was used to trim Illumina adapters and primer sequences (Martin 2017). Amplicon sequence variants were generated and their taxonomic assignments and abundance tables were determined using the package dada2 (v. 1.26.0) (Callahan et al. 2016) in R (v. 4.2.2). Taxonomic classification of the 16S rRNA gene sequence variants was performed using the Silva taxonomic training dataset v138.1 formatted for DADA2 (McLaren 2020). A phyloseq object was created using abundance and taxonomy tables for visualisation of community structure with the package phyloseq (version 1.42.0) (McMurdie and Holmes 2013) in RStudio version 2022.12.0 + 353 (Posit Software, PBC, 2022).

### Quantitative polymerase chain reaction (qPCR)

Separate qPCR analyses targeting the 16S rRNA genes of ammonia-tolerant SPOB, SAOB and methanogens were set up in order to screen for their gene abundance in the flocculating and planktonic communities. To that end, the primers MMBf (5′-ATCGRTACGGGTTGTGGG-3′) and MMBr (5′-CACCTAACGCRCATHGTTTAC-3′) (Yu et al. 2005) targeting the order *Methanomicrobiales* and THACf (5′-ATCAACCCCATCTGTGCC-3′) and THACr (5′-CAGAATTCGCAGGATGTC-3′) targeting the SAOB *Syntrophaceticus* schinkii (Westerholm et al. 2011a, b) were used. For targeting the SPOB ‘*Candidatus* Syntrophopropionicum ammoniitolerans’, novel primers were designed, since existing species-specific primers (Singh et al. 2021) did not encompass all 16S rRNA gene copies of this candidate SPOB, according to the NCBI annotation. Hence, primers SPAf (5′-CCACAGCCTGCCTTTGAAAC-3′) and SPAr (5′-CGTCAGAAACAGGCCAGAGA-3′) were designed as specified previously (Singh et al. 2021). Construction of DNA standard curves was performed as described in Westerholm et al. (2011a, b). The qPCRs were performed in a 20-μL reaction mixture that consisted of 3 μL DNA sample, 10 μL iQ™ SYBR® Green Supermix (Bio-Rad), 1 μL of each primer (10 pmol μL^−1^) and 5 μL UltraPure™ distilled water (Invitrogen). The qPCR protocol for quantification was as follows: 7 min at 95 °C, 40 cycles of 95 °C for 40 s, annealing at 66 °C, 61 °C or 59.3 °C (for the order *Methanomicrobiales*, species *S*. *schinkii* and species ‘*Ca* S. ammoniitolerans’, respectively) for 1 min and 72 °C for 40 s, and melting curve analysis at 95 °C for 15 s, followed by 1 min at 55 °C and finally at 95 °C for 1 s. All reactions were carried out in QuantStudio™ 5 (ThermoFisher).

### Scanning electron microscopy

Samples for SEM were taken from one batch flask of each set of treatment triplicates at the end of A2 and P2. The flasks were opened in an anaerobic box and their rubber stoppers replaced with an adapted rubber stopper with a glass vial inserted in it. To collect undisturbed cultures from the media, the batch flasks were gently tilted to allow the microorganisms to enter the glass vial. The glass vial was then removed from the rubber stopper and closed. Prior to imaging, the samples were filtered (glass fibre filter) with vacuum suction to remove excess liquids. Cell fixation was performed by soaking the filters in a mixture of 4 mL glutaraldehyde, 4 mL phosphate buffer (1 M) and 32 mL deionised water for 4 h, following by soaking in 0.1 M phosphate solution for 10 min. This step was repeated three times, followed by drying with ethanol (50%, 70%, 80%, 90%, 95% and 100% v/v) for 10 min each. The 100% v/v ethanol wash was repeated three times. After the final ethanol drying, the filters were placed in hexamethyldisilazane for 5 min and then left to air-dry in a desiccator overnight. All samples were coated with palladium/gold prior to SEM analysis, which was performed in Geocentrum, Uppsala University, using a Zeiss Supra 35 VP field emission SEM (Carl Zeiss SMT, Oberkochen, Germany) equipped with a variable pressure scanning electron low-vacuum detector and a Robinson backscatter detector. Microphotographs were taken using a Leica MZ75 optical light microscope mounted with a Nikon digital sight DS U1 and NIS-Elements F2.20 software. Images were taken with a beam setting of 4 kV and an aperture of 30.0 µm at an optimal working distance of 8.5 mm. Element analyses were performed using an energy-dispersive X-ray analysis (EDAX) Apex 4 device (Ametekh, Mahwah, USA) coupled with an energy-dispersive X-ray spectroscopy (EDS) detector for X-ray microanalysis.

## Results

### Acetate degradation in the presence of additives

During period A1, the lag phase lasted 49 days for the iron oxide batches and 56 days for all others, after which the added acetate was degraded at relatively similar rates in all sets of batches except those supplemented with graphene, which had a slower degradation rate, though not significant (*p* > 0.5) (Fig. 1a, Table 1). The acetate level decreased to below the detection limit in the iron oxide batches at day 91 and in the other batches at day 98 (Table 1). Measured H_2_ partial pressure varied between 2 and 14 Pa and the pH increased from 7.4 to 8.2 over the course of the experiment (Fig. S1, Fig. S2). The higher pH over time resulted in an increased proportion of free ammonia in the batches (see Table S4 for details), from 0.13 to 0.7 g NH_3_ L^−1^ at the end of A1 (Fig. S3a).

**Table 1:** Lag phase, rate of degradation and day on which acid level was below the detection limit (< 0.2 g L^−1^) in the first and second rounds of acid degradation. Rate of acetate degradation was calculated using values from between days 63 and 84 for A1 and between days 8 and 33 for A2. Rate of propionate degradation was calculated using values from between days 35 and 70 for P1 and between days 44 and 58 for P2. Mean of triplicates and standard deviation are presented, or otherwise mean of duplicates

	First round (A1, P1)			Second round (A2, P2)		
	Lag phase (days)	Rate of degradation (g L^−1^ day^−1^)	Acid below detection (day)	Lag phase (days)	Rate of degradation (g L^−1^ day^−1^)	Acid below detection (day)
Acetate						
Control	56	0.083 ± 0.000	98	8	0.081 ± 0.004	47
Iron oxide (II, III)	49	0.083 ± 0.001	91	8	0.098 ± 0.003^b,c^	40
Graphene	49	0.067 ± 0.010^b^	98	8	0.062 ± 0.009^b^	64
Zeolite	56	0.081 ± 0.004	98	8	0.11 ± 0.012^b,c^	37
Propionate						
Control^a^	28	0.040 ± 0.001	135	36	0.096 0.095	83
Iron oxide (II, III)	28	0.038 ± 0.007	154	27	0.130 ± 0.011^c^	69
Graphene	28	0.048 ± 0.002^b^	119	27	0.100 ± 0.006^c^	135
Zeolite^a^	28	0.037 ± 0.001^b^	147	22	0.095 0.122	58

^a^Standard deviation not calculated in the second round due to loss of one of the triplicate batches

^b^Significant compared to the control of the corresponding round

^c^Significant compared to the correspondent batch during the first round of acid degradation

After feeding with an additional 50 mM of acetate (A2), the lag phase of all batches was 8 days (Table 1). Notably, the iron oxide (*p* < 0.01) and zeolite (*p* < 0.04) batches had significantly faster degradation rates in A2 than in A1, a trend not observed in the graphene and control batches. Hence, in A2, the acetate levels fell below the detection limit after 37 and 40 days for the iron oxide and zeolite batches, respectively, and after 47 and 64 days for the control and graphene batches, respectively (Fig. 1b, Table 1). The SAO community showed a significantly faster acetate degradation rate in the presence of iron oxide (*p* < 0.02) and zeolites (*p* < 0.04) than in the control, while the presence of graphene significantly hampered acetate degradation rate (*p* = 0.049).

### Propionate degradation in the presence of additives

During period P1, all SPO-enriched batches had a lag phase of 28 days (Table 1). All batches displayed similar propionate degradation profiles, consisting of two phases of linear degradation (between days ~ 30–70 and 100–120/150). At the point at which acetate level began to decrease (day 70 for the controls, day 63 for all other batches), a phase of slower propionate degradation was observed (days ~ 70–100), i.e. when propionate and acetate level was around 1.5–2 g L^−1^ and 1.2–1.5 g L^−1^, respectively (Fig. 2a and c). During this intermediary lag phase, H_2_ partial pressure increased from 3.5–5 to 5.5–7.5 Pa (Fig. S4) and pH increased from 7.6 to 7.7–7.9 (Fig. S5). The latter resulted in temporary increased ammonia level (see Table S4 for details), from 0.2 to 0.3–0.4 g L^−1^ (Fig. S3b). A phase of faster propionate degradation coincided with lowering of the ammonia concentration (from 0.4 to 0.2 g L^−1^) and H_2_ level (from 6–7 to 1–3 Pa), where they remained stable until the end of the experiment. The molar conversion ratio of propionate into acetate did not reach 1:1 at the peak acetate level (except for the graphene batches), indicating that the SAO community was active and degraded acetate concurrently with its formation (Fig. S6). However, the accumulation of acetate up until days 63–70 indicated that acetate degradation by the SAOB was not sufficiently fast to match the rate of propionate degradation by the SPOB. Following the peak in acetate levels, both acetate and propionate were consumed until they dropped below the detection limit.

Regarding the impact of additives on propionate degradation, inclusion of graphene had a significant (*p* = 0.005) positive impact on the degradation rate during P1, whereas the presence of iron oxide did not have a significant effect (*p* > 0.06) and the zeolite significantly (*p* < 0.05) lowered the degradation rate (Table 1, Fig. 2a). Accordingly, in the presence of graphene, propionate was degraded to below the detection limit after 119 days, whereas the batches with other amendments required 135–154 days to deplete all propionate. The acetate level in all sets of batches was below the detection limit before day 105 (Table 1, Fig. 2a and c). The H_2_ partial pressure during P1 varied between 1 and 7 Pa (Fig. S4), which was somewhat lower than in A1. The pH varied between 7.4 and 7.9 in all batches during P1, which was a lower pH increase than in the acetate-fed batches (Fig. S5). This resulted in the concentration of ammonia increasing from 0.12 to 0.32 g NH_3_ L^−1^ at the end of P1 (Fig. S3b).

In contrast to the acetate experiment, the lag phase in P2 was similar to that in P1, varying between 22 and 36 days (Table 1). Interestingly, a different degradation curve to that in P1 was observed after the second feeding of propionate. During P2, all batches exhibited a single faster degradation phase (Fig. 2b) and a higher rate of propionate consumption than in P1, which was highest for the iron oxide batches, followed by the zeolite batches, and for the graphene and control batches, which had similar degradation rates (Table 1). These differences were statistically significant (*p* < 0.001) for both iron oxide and graphene batches, while for the control and zeolite batches the significance of these differences was not possible to determine. Throughout P2, acetate concentration built up to a peak at around 2.3–2.8 g L^−1^, after which it was steadily consumed to below the detection limit (Fig. 2d). The peak approximately coincided with the time point at which the propionate level was below the detection limit for the iron oxide and zeolite batches, while for the control and graphene batches, the point at which acetate level began to decrease (day 79 for the controls, day 76 for the graphene batches), a phase of slower propionate degradation was observed (Fig. 2b and d). In P2, the acid levels in the zeolite batches were below detection after 80 days, whereas in the other batches acids were fully consumed at between 111 and 135 days (Table 1, Fig. 2b and d).

Cultivation experiments conducted to investigate the impact of acetate on propionate degradation rate and lag phase in the absence of additives showed that presence of acetate up to 2 g L^−1^ had a low impact on propionate degradation rate of the SPO enrichment culture during degradation of 3.5 g L^−1^ propionate (Fig. S7). However, in the presence of 4 g L^−1^ acetate, propionate was degraded at a reduced rate. In the SPO culture initiated at 7.5 g L^−1^ propionate, acetate concentrations of 1 g L^−1^ and above decreased the propionate degradation rate (Fig. S7).

### Methane formation and carbon balance calculations

In A1, 22–24 mmol of methane was formed, corresponding to a yield of 88–96% based on the carbon balance calculations (Fig. S8a). In A2, all batches produced 18–20 mmol of methane. Hence, about 72–80% of the acetate present was converted to methane in A2, which was significantly (*p* < 0.01) lower than in A1 (Fig. S8b). During P1, the carbon balance calculations demonstrated equimolar conversion of 25 mmol propionate into methane (39–40 mmol, i.e. 98–100% yield) (Fig. S9a). In P2, 30–31 mmol of methane was produced, which represented a significantly (*p* < 0.01) lower conversion yield for all batches (75–78%) in comparison with P1 (Fig. S9b).

### Microbial community structure

**Fig. 3:**
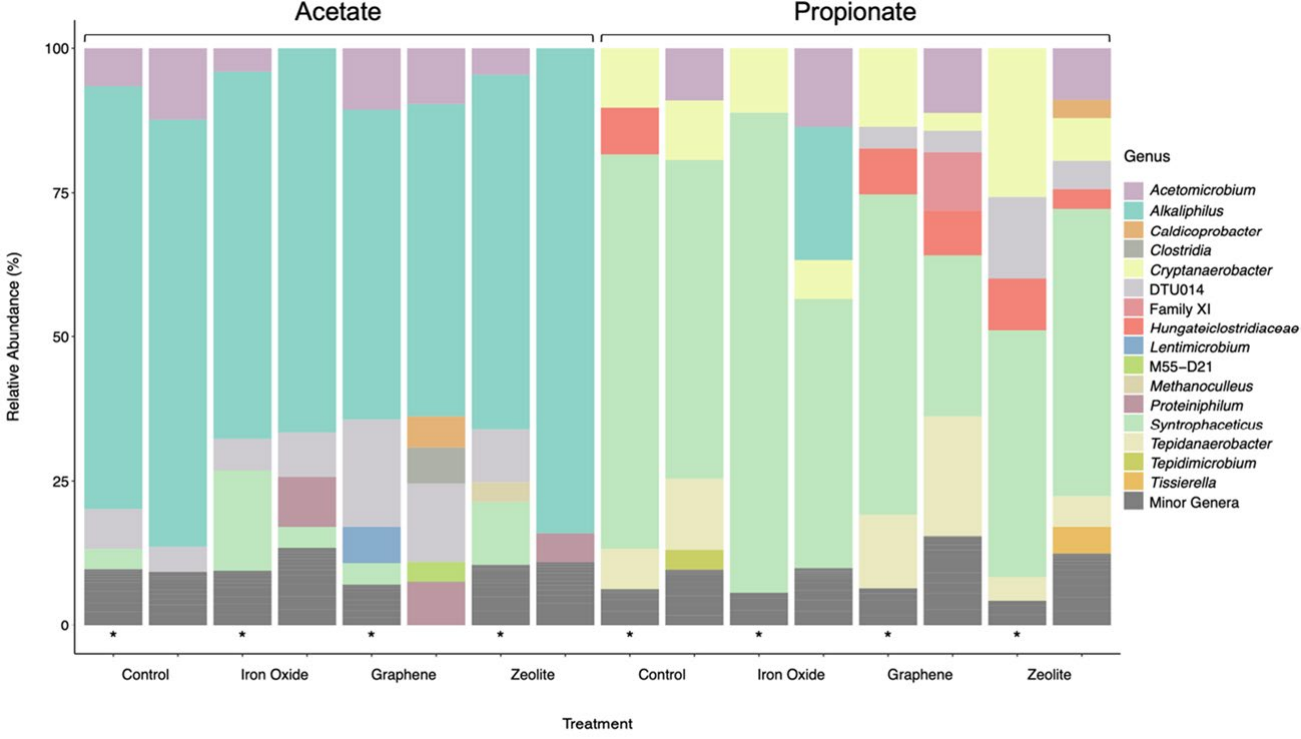
Microbial community structure at genus level in the flocculating (bars marked with an asterisk) and planktonic communities in the acetate- and propionate-fed batches. All samples were taken at the end of the second round of acid degradation (A2, P2). The threshold for representation in the image was 3%. The sequence assigned as *Cryptanaerobacter* was 99% similar to the 16S rRNA gene sequence of ‘*Ca* S. ammoniitolerans’ (Singh et al. 2021)

Analysis of microbial community composition by Illumina sequencing targeting the 16S rRNA gene revealed differences in community composition, particularly between the acetate and propionate experiments, but also between the flocculating and planktonic microbial communities (Fig. 3). In the acetate-fed batches, the most abundant genus was *Alkaliphilus*, with relative abundance varying from 53 to 74% in all batches and with no discernible differences between flocculating and planktonic communities. Another species present in all batches was the SAOB *S. schinkii* (99% sequence similarity based on 16S rRNA gene sequence). Interestingly, this SAOB was present in higher relative abundance in the flocculating communities (3–17%) than in the planktonic communities (< 3–4%), although the difference was not statistically significant (*p* = 0.073) (Fig. 3). Members of the unclassified DTU014 and the genus *Acetomicrobium* were also detected (< 3–25%) in all communities except the planktonic communities in the zeolite and iron oxide batches.

In the propionate-fed batches, the predominant species in all samples was *S. schinkii* (Fig. 3). Again, this species was present in higher relative abundance in the flocculating communities (43–83%) than in the planktonic communities (28–55%), but the difference was not significant (*p* = 0.066). Another genus present in all propionate-fed batches was *Cryptanaerobacter*. Sequence comparison revealed this to be ‘*Candidatus* Syntrophopropionicum ammoniitolerans’, previously identified as a candidate SPOB in the reactors from which the inoculum originated (99% similarity based on sequence alignment of the 16S rRNA gene retrieved from MAG62 in Singh et al. (2021), accessible at GenBank JABMJD000000000, hereafter referred to as ‘*Ca* S. ammoniitolerans’) (Singh et al. 2021). This candidate SPOB was present at higher relative abundance in the samples taken from flocs (10–26%) than in those taken from planktonic microorganisms (3–10%), although this difference was not statistically significant (*p* = 0.058). Furthermore, a species previously known as an ammonia-tolerant SAOB, *Tepidanaerobacter acetatoxydans* (Westerholm et al. 2011a, b) (96% similarity based on nucleotide sequence blast of the 16S rRNA gene), was present above the detection threshold in all propionate-fed batches except the iron oxide batches. The relative abundance of this species was 4–13% in the flocculating communities, whereas it represented 5–21% of the planktonic communities (Fig. 3). Also present at higher relative abundance in the flocculating communities (< 3–16) than in the planktonic communities (< 3–10%) (except in the graphene batches) was the family *Hungateiclostridiaceae*. In the zeolite batches, the unclassified DTU014 was detected at higher relative abundance in the flocculating community (3–30%) than in the planktonic community (4–6%). Another notable finding was higher relative abundance of *Acetomicrobium* in the planktonic community (3–26%) than in the flocculating community (< 3–5%) in the propionate-fed batches (Fig. 3). Relative abundance of a methanogenic partner only exceeded the threshold in the zeolite-amended, acetate-fed flocculating community. From this sample, genes affiliated to the methanogenic genus *Methanoculleus* were obtained.

### Quantitative abundance of SPOB, SAOB and hydrogenotrophic methanogens in flocculating and planktonic communities

**Fig. 4:**
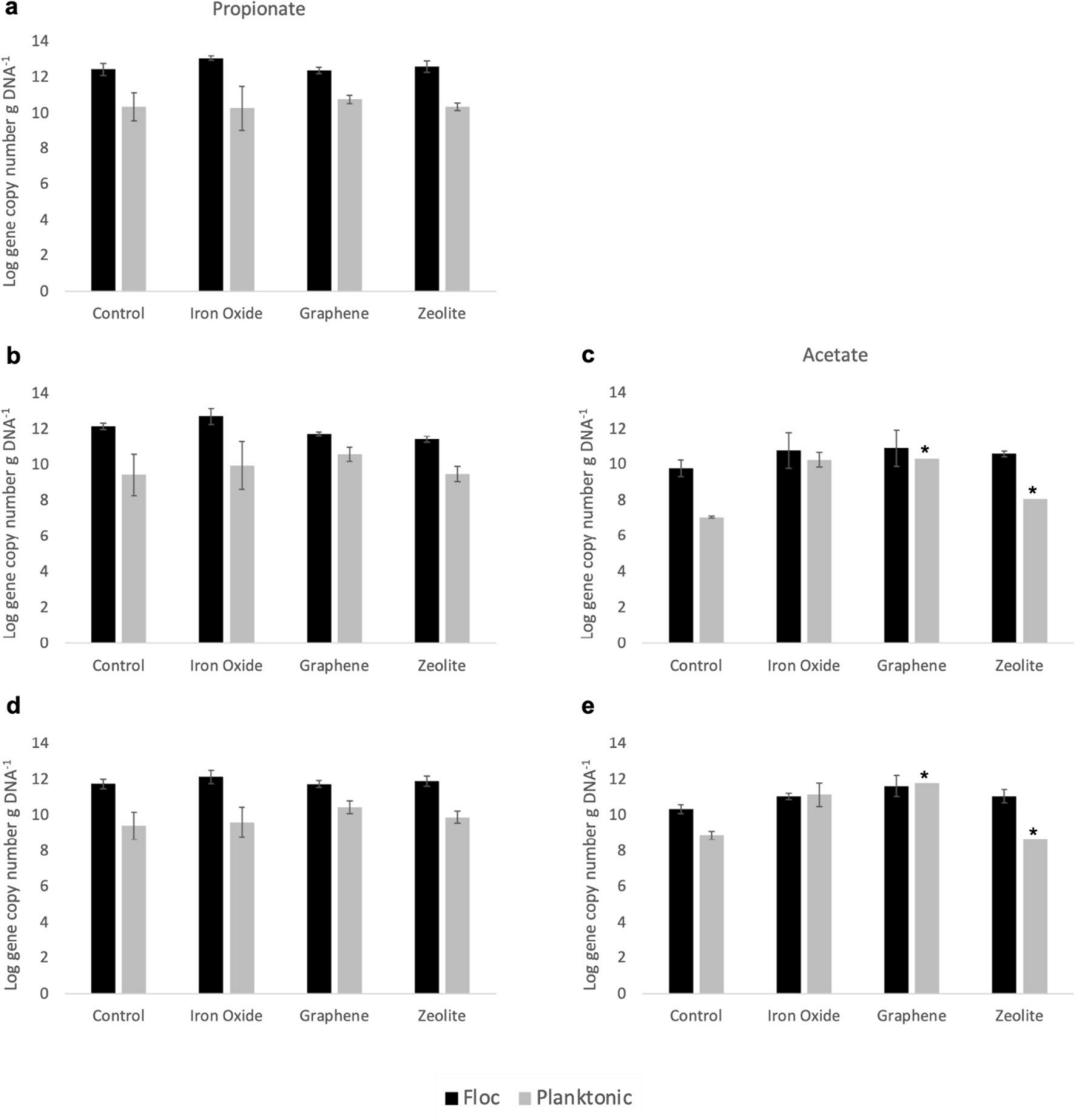
Gene copy number of **a** the syntrophic propionate-oxidising bacteria ‘*Ca* S. ammoniitolerans’, **b**, **c** the syntrophic acetate-oxidising bacteria *S. schinkii* and **d**, **e** the partner hydrogenotrophic methanogen in the flocculating and planktonic communities of the propionate (left panels) and acetate (right panels) degradation experiments. All values are mean of triplicates except those marked with and asterisk, which are mean of duplicates

In the SPO enrichment cultures, quantification of methanogens using qPCR revealed significantly higher levels of hydrogenotrophic methanogens belonging to the order *Methanomicrobiales* in the flocculating communities than in the planktonic communities in all batches (*p* < 0.01). In the SAO enrichment cultures, only the control had significantly higher levels of methanogens in the flocculating communities (*p* < 0.02) than in the planktonic communities, while the batches treated with iron oxide had higher levels in the planktonic communities than in the flocculating communities (difference not significant; *p* > 0.8) (Fig. 4d and e). The graphene- and zeolite-amended batches had, respectively, lower and higher methanogen levels in their flocculating communities than in planktonic communities, but the significance of these differences could not be confirmed. Similarly, the SAOB *S. schinkii* was present in higher levels in flocculating communities than in planktonic communities in the acetate-fed, graphene- and zeolite-amended batches, but this difference could not be statistically verified. However, the zeolite batches of the acetate-fed cultures demonstrated significantly (*p* < 0.05) higher levels of this SAOB in their flocculating communities than the control batches. This SAOB was also present in significantly (*p* < 0.01) higher levels in flocculating communities than in planktonic communities of the control, but not of the iron oxide batches (*p* > 0.5), in the acetate experiment (Fig. 4c). In the propionate-fed batches, this SAOB was present in significantly higher levels in the flocculating communities than in the planktonic communities of all batches (*p* < 0.03), with the flocculating communities of both the graphene- and zeolite-amended batches displaying significantly (*p* < 0.03) higher SAOB levels than those of the control (Fig. 4b). The SPOB ‘*Ca* S. ammoniitolerans’ was also present in significantly higher levels in flocculating communities than in planktonic communities of the propionate-fed batches (*p* < 0.02) (Fig. 4a). For this organism, only the flocculating communities of the iron oxide batches had significantly higher values than the control (*p* < 0.05).

### Scanning electron microscopy

**Fig. 5:**
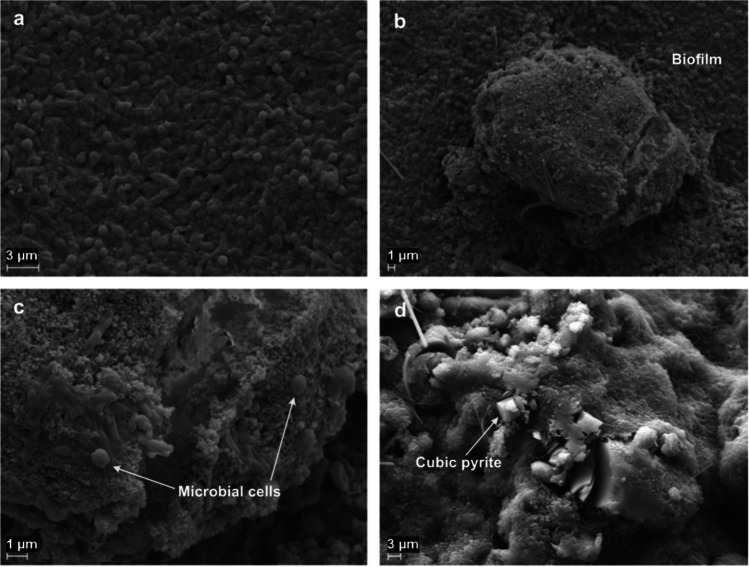
SEM images of the cultures supplemented with iron oxide. Images **a**–**c** were obtained from propionate-degrading cultures, while image **d** was obtained from acetate-degrading cultures

The SEM images of samples from the iron oxide-supplemented batches showed densely packed microbial communities attached to the iron oxide grains (Fig. 5a–c). Element analyses using EDAX and EDS demonstrated presence of pyrites (FeS_2_, cubic) as well as phosphate precipitates (vivianite) in both the acetate- and propionate-degrading batches supplemented with iron oxide nanoparticles (Fig. 5d).

**Fig. 6:**
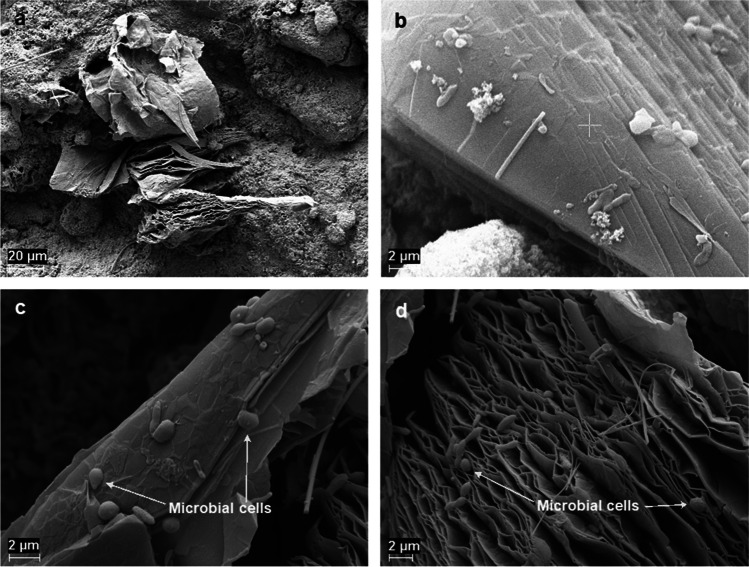
SEM images of the cultures supplemented with graphene. Images **a** and **b** were obtained from propionate-degrading cultures, while images **c** and **d** were obtained from acetate-degrading cultures

In contrast to the extensive surface colonisation visible in the samples from iron oxide-supplemented batches, the images of the acetate- and propionate-fed cultures with added graphene showed few scattered cells attached on the grain surface and attachment of cells in between the graphene grains (Fig. 6). No extensive phosphate precipitation or other secondary minerals were detected in the samples from the cultures with graphene addition.

**Fig. 7:**
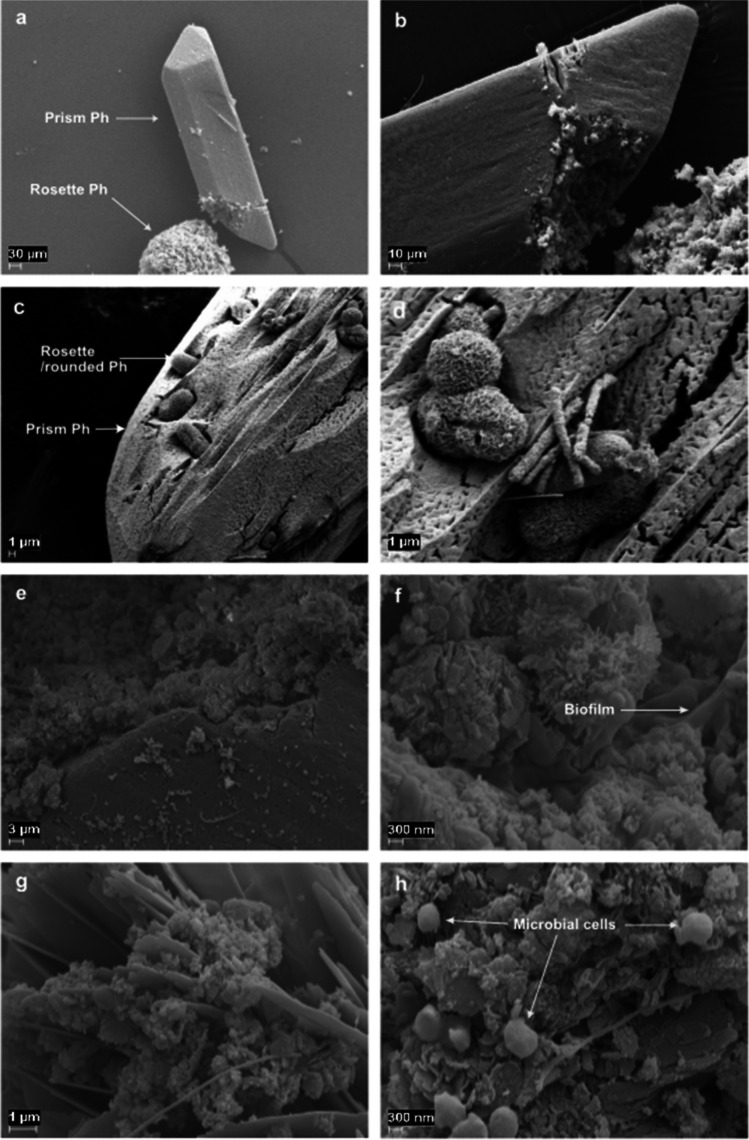
SEM images of the cultures supplemented with zeolite. Images **a**–**d** were obtained from acetate-degrading cultures, while images **e**–**h** were obtained from propionate-degrading cultures

All SEM images of the cultures supplemented with zeolite showed extensive precipitation of phosphates (Ph) of different crystal morphologies (Fig. 7a–h). In the sample from propionate-degrading cultures, prism-like (Fig. 7a–e), rosette-like rounded (Fig. 7a, c, d and g) and irregular (Fig. 7e, h) calcium and magnesium phosphates were visible. The microorganisms colonised the surface and formed biofilm on all types of phosphate precipitates (Fig. 7h). The EDAX and EDS analyses revealed no precipitates other than phosphates in the zeolite cultures.

## Discussion

### Propionate and acetate degradation dynamics and carbon balance were altered after second addition of acids

Irrespective of the presence of additives, all propionate-degrading batches showed faster acid consumption during the second substrate degradation (P2) compared with the first (P1), while the acetate batches had a shorter lag phase in the second substrate degradation (A2) compared with the first (A1) (Figs. 1 and 2, Table 1). This is likely explained by the visibly higher abundance of microorganisms and flocs (Fig. S10c and b) and by metabolically important enzymes having already been formed at this point, but not at the beginning of A1/P1 (Fig. S10a and b) (Thiele et al. 1988; Brock et al. 2003). Another conceivable explanation is that the higher pH levels (7.9/8.2) observed at the beginning of the second round of degradation may have been more conducive to growth of syntrophic communities than the lower pH (7.3) prevailing at culture initiation. While the optimal pH for these enrichment cultures remains undetermined, an optimum range around 7.9–8.2 would align with conditions typically found in high-ammonia biogas processes (Westerholm et al. 2015, 2020; Fischer et al. 2019), from where the inoculum cultures used in these experiments were taken. Notably, higher pH increased the ammonia level in the medium, which commonly lowers overall microbial activity (Yenigün and Demirel 2013). However, performance studies conducted with the main SAOB used in the present work have shown that even at elevated ammonia levels up to 1.8 g L^−1^ (at pH 8.1 and 38 °C) methane formation is not impeded, but rather accelerated (Westerholm et al. 2019). Moreover, previous studies have found that the main SAOB in this study, *S. schinkii*, cannot grow as pure culture at pH 8.5 (Westerholm et al. 2010). However, it has been shown that the growth parameters for organisms in pure culture can differ from those in co-culture (Hanly et al. 2012) and that formation of flocs (where these syntrophic microorganisms were found in higher abundance) can also provide protection against adverse conditions such as alkaline pH (Charles et al. 2017) and can increase tolerance to inhibitors (Westman et al. 2014).

The biphasic propionate degradation wherein acetate accumulated and underwent oxidation prior to ultimate degradation of propionate observed in P1 aligns with a previous study of the enriched mesophilic SPOB community used in the present study (Weng et al. 2024). In that study, thermodynamic calculations indicated low impact by acetate levels on the propionate degradation. Similarly, our results showed that propionate degradation by the SPO enrichment culture (initiated at 3.5 g L^−1^ propionate) was not impaired by acetate levels at or below 2 g L^−1^ (Fig. S7). An alternative explanation is that the onset of an accelerated phase of acetate degradation altered the environmental conditions, resulting in a rise in H_2_ levels and in pH, and thus in ammonia levels (Fig. S3b, Fig. S4 and Fig. S5), temporarily impeding SPOB activity. Notably, a similar biphasic propionate degradation was not observed in P2, possibly because propionate was degraded faster and already completely consumed at the point of initiation of acetate degradation. However, in the control and the graphene batches in P2, the relatively slower degradation rate of propionate left approximately 0.2–0.3 g L^−1^ of propionate in the cultures when syntrophic acetate oxidation began. Consequently, this minor amount of propionate was not degraded until acetate was depleted, which happened after about 140 days of incubation (Fig. 2). Another interesting finding of the present study was that, unlike the acetate cultures, P2 did not show a shorter lag phase in any of the batches in comparison with P1. Syntrophic propionate oxidation has a notoriously long lag phase due to its energetically unfavourable reactions (Imachi et al. 2000, 2002). Metagenomic studies have indicated that, like several other SPOB (Kato et al. 2009; Hidalgo-Ahumada et al. 2018), ‘*Ca* S. ammoniitolerans’ connects the first step of endergonic propionate activation with the last step of exergonic acetyl-CoA de-activation (Singh et al. 2021). This can explain the long lag phase in initial stages of propionate degradation and after a starvation period, i.e. at the beginning of P1 and P2.

In A1 and P1, methane yield from conversion of acetate or propionate corresponded to 88–100% of the expected methane production (Fig. S8, Fig. S9) based on the stoichiometry of the oxidation reactions (Table S3). This yield range is in line with values reported in previous studies on syntrophic propionate and acetate oxidation under high-ammonia and thermophilic conditions (Singh et al. 2023) and on syntrophic acetate oxidation under high-ammonia and mesophilic conditions (Westerholm et al. 2019). However, the significantly lower levels obtained in A2 and P2 (72–80% of expected methane production; Fig. S8, Fig. S9) given the existing biomass are puzzling. Further research into the carbon and energy balance of catabolic and anabolic reactions is required to explain this finding.

### Additives affected microbial colonisation, floc formation and propionate and acetate degradation rates

The acetate and propionate enrichment cultures revealed some similar and some diverging impacts of the different additives on the degradation rate. Zeolite and iron oxide had positive effects on the degradation rate by the SAO (A2) and SPO enrichment cultures (P2). These were the two fastest batches to degrade acetate in both A2 and P2 (Figs. 1b and 2b), while during P1, both treatments promoted faster degradation of acetate, preventing these cultures from reaching a 1:1 ratio between propionate degradation and acetate formation, unlike in the control batches and particularly the graphene batches (Fig. S6). These results are in agreement with previous reports of positive effects of addition of magnetite (Fe(II,III)_3_O_4_) (Viggi et al. 2014; Wang et al. 2018a, b) and zeolites (Milán et al. 2001; Tada et al. 2005) on VFA degradation in anaerobic processes under high-ammonia conditions. The rates of acetate and propionate degradation observed in the present study were comparable to those in other studies degrading complex materials. For instance, in a previous study using sludge from a wastewater treatment facility as inoculum and an ammonia level of 5 g L^−1^ NH_4_Cl, cultures with iron oxide nanoparticles had acetate degradation rates of approximately 0.075 g L^−1^ day^−1^ (Zhuang et al. 2018), while in the present study the rate in iron-oxide-amended cultures was 0.098 g L^−1^ day^−1^ for A2. Another study investigating the impact of iron oxide nanoparticles on degradation of propionate under low-ammonia conditions (0.5 g L^−1^ NH_4_Cl) using an inoculum from waste-activated sludge observed propionate degradation rates of approximately 0.029 g L^−1^ day^−1^ after a second propionate feeding (Viggi et al. 2014). In the present study, the iron oxide-amended batches degraded propionate at a rate of 0.130 g L^−1^ day^−1^ after a second propionate feeding (P2). Overall, our results indicated higher rates of propionate and acetate degradation in enriched syntrophic cultures supplemented with iron oxide nanoparticles than in sludge inoculum supplemented with these acids in previous studies. These differences could be related to promotion of syntrophic interactions in the enrichment cultures by the iron oxide nanoparticles, whereas in more complex cultures this material may influence other microbial interactions than the syntrophic cooperation (Sarker and Nikhil 2023). Regarding the positive effect of zeolite on acetate (A2) and propionate (P2) degradation demonstrated in the present study, some studies degrading complex substrates (food waste) have been performed under ammonia levels around 0.3 g NH_3_ L^−1^, at which some ammonia stress can be observed (Cardona et al. 2021). However, little information is available on acetate and propionate degradation by highly enriched syntrophic cultures, highlighting the novel value of our study.

Conductive additives have been suggested to enable DIET between microorganisms lacking e-pili or multiheme c-type cytochromes (Liu et al. 2012, 2015), which could be another possible explanation for the faster degradation rate in the iron oxide cultures in A2 and P2. The extensive colonisation of iron oxide nanoparticles (Fig. 5a–c), zeolites (Fig. 7e, h) and precipitates in these cultures suggests that the microbial community benefited from their presence as support structures. These additives may facilitate establishment of interspecies connections and short-circuit the electron transfer, either through DIET or through diffusion of intermediary compounds such as formate and H_2_, or possibly through co-occurrence of both processes (Thiele and Zeikus 1988; Dolfing 2013). Zeolites may have contributed to the enhanced degradation rates observed in A2 and P2, potentially due to their content of loosely bound surface cations (e.g. Ca^2+^ and Mg^2+^). When exchanged with other ions present in the solution (Pabalan and Bertetti 2001), such as NH_4_^+^, these cations could have promoted the capability for acid degradation in the culture. This could have alleviated the ammonia stress (Wang and Peng 2010), while the slightly elevated Ca^2+^ and Mg^2+^ concentrations within the solution could also have stimulated microbial activity (Wang et al. 2023). For instance, Ca^2+^ is an important regulator that can influence bacterial cell structure, differentiation and gene expression (Smith 1995) and biofilm formation, regulating the diffusion of fluids, inorganic and organic compounds and toxic molecules (Keren-Paz and Kolodkin-Gal 2020). Furthermore, Mg^2+^ is a cofactor for a wide range of enzymes that stabilise nucleic acids and ribosomes, for ATPases and for maintaining cell integrity (Smith and Maguire 1998).

The formation of pyrites (FeS_2_, cubic) in the iron oxide-supplemented acetate- and propionate-degrading cultures (Fig. 5d) indicated that the additional available iron altered the chemical conditions for the syntrophic communities. The basal medium used in the present study had redox potential of − 330 mV and initial pH of 7.3, which is within the solid stability field of the Fe-S system of FeS and possibly FeS_2_ (Lyon 2010). The basal medium also had a Fe concentration of 10^−6^ M and S concentration of 10^−3^ M, and was thus already saturated with respect to formation of iron sulphides, such as cubic FeS_2_ and tetragonal layered FeS. Hence, in the iron oxide-supplemented cultures, the increase in pH to 7.9–8.2 during growth most likely promoted formation of cubic FeS_2_. The initial pH in the cultures could have enabled formation of tetragonal layered FeS, but only cubic FeS_2_ was observed in the samples. Nevertheless, FeS is within the stability field and may therefore also be present in the samples. To scan the solid phase for secondary minerals, further SEM–EDS mapping or X-ray diffraction analysis would be required. Vivianite $\left({{\mathrm{Fe}}_3}^{2+}{\left({\mathrm{PO}}_4\right)}_2\cdot8{\mathrm H}_2\mathrm O\right)$ precipitates were also formed in the iron oxide-supplemented cultures. Formation of this precipitate when phosphates and Fe(II,III)_3_O_4_ are present in the medium has been reported previously (Hao et al. 2022; Prot et al. 2022). Our element analyses also demonstrated formation of an extensive amount of phosphate precipitates in the zeolite-supplemented cultures. The impact of formation of these precipitates on acid degradation rate is still unclear and further research is required, but several studies have shown that phosphates in solution can reduce methanogenic activity under low-ammonia conditions and hinder consumption of acids such as acetate, propionate and butyrate (Paulo et al. 2005; Lackner et al. 2020). However, it was not possible to assess the effect of phosphate dissolution or precipitation on acid consumption and methanogenesis in the present study.

Interestingly, graphene had a positive effect on propionate degradation rate in the SPO enrichment cultures, but not on acetate degradation in the SAO enrichment cultures. The positive effect in P1 and P2 could have the same explanation as suggested for the effect of iron oxide nanoparticles in A2 and P2, as graphene itself is a conductive material that can permit DIET (Florentino et al. 2019; Zhou et al. 2022). It has even been shown that addition of graphene to a biogas process degrading food waste can promote e.g. lower propionate accumulation and shorter culture lag phase (Capson-Tojo et al. 2018). However, the impaired acid degradation in both A1 and A2 is intriguing and supports the higher acetate accumulation resulting from propionate degradation in P1, in the graphene-amended batches. The reason for this negative impact is not clear, but it has been suggested that carbon nanoparticles can interfere with cell membrane integrity, thereby compromising cell viability and metabolic capacity (Akhavan and Ghaderi 2010; Zhou and Gao 2014). Previous studies in the degradation of sludge fed with acetate had already shown that acetate degradation was not positively affected by addition of granular activated carbon (Xu et al. 2018). Moreover, the colonies of microorganisms in between the graphene grains were not as extensive as in the iron oxide- and zeolite-amended cultures (Fig. 7b–d and f–h), where the entire surface of the additives was covered by microbial colonies, and phosphate precipitates were not formed in these samples. These results indicate that graphene addition promoted biofilm formation to a lesser extent than the other additives. This can help explain the lower rate of acid degradation in the SAO enrichment cultures and the accumulation of acetate in P1 in the graphene-amended cultures compared with the iron oxide and zeolite cultures, since syntrophic metabolism relies on physical proximity of the acid oxidisers and their partner methanogen (Cord-Ruwisch et al. 1998; Felchner-Zwirello et al. 2013). However, the apparent similar rate of acetate degradation after peaking acetate values during P1 and P2 remains enigmatic.

### Higher abundance of SPOB, SAOB and methanogens in flocculating communities reflects high importance of cell proximity for syntrophic activities

The relative microbial abundance profile in the SAO enrichment cultures indicated that over half of the microbial community at the end of A1 comprised members of the genus *Alkaliphilus*. This genus has previously been suggested to include potential ammonia-tolerant SAOB (Mosbaek et al. 2016; Westerholm et al. 2018), but this has not yet been proven. It should be noted that *Alkaliphilus* sp. has the ability to grow on several substrates, such as the yeast extract added to the medium in the present study (Takai et al. 2001; Zhilina et al. 2009). In contrast, *Alkaliphilus* sp. was not detected at high relative abundance in our SPO enrichment cultures, except for the planktonic iron oxide communities (Fig. 3). Members of *Alkaliphilus* are alkaliphilic and have a pH optimum between 7.5 and 10 (Takai et al. 2001; Cao et al. 2003; Fisher et al. 2008), which coincides with the higher pH in the SAO cultures, while the lower pH in the SPO cultures would be less favourable for the *Alkaliphilus* member present in the SAO cultures. *Syntrophaceticus schinkii* was present in high relative abundance in both the SAO and SPO enrichment cultures. This species is a known SAOB (Westerholm et al. 2010), suggesting a significant role as the primary acetate-degrading species in the SPO enrichment cultures and very likely also in the SAO enrichment cultures. The qPCR results showed that *S. schinkii* was present in higher abundance in flocculating communities than in planktonic communities across all SPO and SAO enrichment cultures, alongside their partner methanogen (this latter with the exception of the acetate-fed, iron oxide- and graphene-amended batches). This strongly indicates that these syntrophic microorganisms strive for physical proximity to facilitate their syntrophic metabolism. Interestingly, zeolite addition seemed to promote recruitment of *S. schinkii* in both the acetate- and propionate-fed batches, which had significantly higher levels of this SAOB than the control (*p* < 0.05), while graphene seemed to promote its presence in the propionate-fed batches (*p* < 0.03). As found in a previous SAO enrichment study (Westerholm et al. 2019), another known SAOB, *T. acetatoxydans*, was present at considerably lower relative abundance than *S. schinkii*, in particular in the flocculating communities. In fact, its relative abundance in the acetate-degrading enrichments was even lower than 3% in all batches. This suggests that *T. acetatoxydans* was a secondary SAOB in both the acetate- and propionate-degrading enrichment cultures and did not play such an important role in the flocculating communities.

As with the SPOB, the high relative abundance of ‘*Ca* S. ammoniitolerans’ in the propionate-fed batches (and its absence in the SAO enrichment cultures) indicated that it was the main SPOB in the propionate-degrading enrichment cultures. This species has previously been identified as a candidate SPOB in the reactors from which the inoculum used in the present study originated (Singh et al. 2021). The qPCR results confirmed that this SPOB was present in significantly higher abundance in flocculating communities than in planktonic communities (*p* < 0.02), further stressing the importance of these syntrophic microorganisms living in close proximity to complete their metabolism. The qPCR results indicated that iron oxide addition promoted recruitment of this SPOB to flocculating communities, where it displayed significantly higher levels than in control batches (*p* = 0.04). Another relevant finding is presence of the genus *Acetomicrobium* in the planktonic samples. Activity of members of this genus has been identified previously in the mesophilic community used in the present study (Weng et al. 2024) as well in acetate- and propionate-fed reactors under thermophilic conditions (Singh et al. 2023). It has been hypothesised that this species might be involved in formate utilisation or conduct syntrophic acetate oxidation, but the species is also described as capable of growing on a broad spectrum of substrates, including yeast extract and cysteine (Menes and Muxí 2002; Hania et al. 2016) which is present in the medium used in the present study.

As found in several previous microbial studies with syntrophic cultures and high-ammonia conditions (Westerholm et al. 2010, 2019; Maus et al. 2015; Manzoor et al. 2016), a member of the genus *Methanoculleus* was the main partner methanogen for the syntrophic bacteria. *Methanoculleus bourgensis* has previously been found to be the partner methanogen in co-culture with *S. schinkii* (Westerholm et al. 2019), whereas the partner methanogen to the SPOB and SAOB in enrichment cultures in the present study has previously been identified to be a novel *Methanoculleus* species, with the provisional name ‘*Candidatus* Methanoculleus ammoniitolerans’ (Weng et al. 2024). The qPCR results showed that hydrogenotrophic methanogens were also present in significantly higher abundance in the flocculating communities than in the planktonic communities (*p* < 0.01), with the exception of the SAO enrichment cultures treated with iron oxide (*p* > 0.8) and graphene. It is worth pointing out that no other group of methanogens was present in relative abundance of at least 1%, in agreement with findings in other studies conducted on anaerobic digestion under high-ammonia conditions (Schnürer and Nordberg 2008; Fotidis et al. 2014; Westerholm et al. 2015). To sum up, a significant increase in abundance of certain syntrophic microorganisms within flocculating communities compared with planktonic communities was observed. Specifically, the SPOB ‘*Ca* S. ammoniitolerans’ and SAOB *S*. *schinkii* showed higher abundance within flocculating communities in all propionate-fed enrichment cultures (*p* < 0.03), with iron oxide addition significantly promoting the recruitment of the SPOB (*p* = 0.04), and graphene and zeolite addition significantly promoting the recruitment of the SAOB to these communities (*p* < 0.03). Furthermore, *S*. *schinkii* was present in higher abundance within flocculating communities in all acetate-fed enrichments. Additionally, there was notably higher abundance of *Methanoculleus* sp. in all propionate- and acetate-fed enrichment cultures (*p* < 0.01) except for the acetate-fed enrichments amended with iron oxide or graphene. These results are in accordance with previous studies (Cheng and Call 2016; Wang et al. 2019) and suggest that the impact of additives may derive not only from promotion of syntrophic activity, but also from number of syntrophic microorganisms recruited in flocs.

To conclude, addition of iron oxide nanoparticles and zeolite enhanced the rate of both acetate and propionate degradation, following a second acid addition. Both additives also promoted microbial colonisation of their surfaces, enabling close proximity between microbial cells that likely facilitated faster acid consumption. Addition of graphene impaired the rate of acetate degradation and did not promote microbial colonisation of its surface but promoted propionate degradation. Microbial community composition and abundance of syntrophic bacteria and associated methanogens differed markedly between flocs and planktonic samples. The main genus present in acetate cultures was *Alkaliphilus* sp., followed by the SAOB *S. schinkii*, which was found in higher abundance in flocs than in planktonic communities, except in acetate-fed, iron oxide- and graphene-amended cultures. In the propionate cultures, the candidate SPOB was identified as ‘*Ca* S. ammoniitolerans’, while the SAOB was *S. schinkii*. Both these species were present in significantly higher abundance in flocs than in planktonic communities in all propionate-degrading cultures. In both acetate and propionate cultures, the partner methanogen was ‘*Ca*. *Methanoculleus* ammoniitolerans’, which was present in significantly higher abundance in flocs than in planktonic communities in all propionate cultures and in the acetate control cultures. Furthermore, the cultivation experiments with different acetate levels revealed that the acetate concentration per se had minimal influence on propionate degradation. Nevertheless, the outcome implied that the two syntrophic bacteria (the SPOB and the SAOB) encountered challenges in maintaining simultaneous activity.

Novel approaches that enhance SAOB and SPOB abundance and activity in biogas reactors will result in better reactor performance in high-ammonia biogas processes, by preventing VFA accumulation and lowered methane yield, and a risk of complete system failure. Addition of conductive and non-conductive additives such as the ones used in this study can stimulate syntrophic metabolism by aiding floc formation, precipitating or removing compounds known to inhibit different steps of the anaerobic digestion process, or by providing nutrients which are required by these microorganisms to fulfil their metabolisms. While graphene may not be an additive to consider for an industrial-scale biogas digester due to its cost, iron oxide particles and zeolites are inexpensive, readily available additives with good prospects of being applied in industrial-scale biogas digesters operating under high-ammonia conditions. Further experiments focusing on the suitability of such additives for different types of reactors and operating conditions (type of substrate, temperature, pH, ammonia levels, etc.) and on the optimisation of concentration of additives at small-scales (to allow validation of laboratory-scale results and troubleshooting of possibly emerging operational problems) should then be conducted before industrial implementation. It is therefore of critical importance for biogas production processes to address ammonia inhibition through identification and inclusion of suitable additives, evaluation of their feasibility of use and optimisation from a functional, economic and environmental standpoint.

## Supplementary Information

Below is the link to the electronic supplementary material.

Supplementary file1 (PDF 3.39 MB)

## Data Availability

The raw sequencing data obtained in this study are available at NCBI under BioProject ‘Impact of supportive materials on syntrophic propionate and acetate enrichments under high-ammonia’ (PRJNA1069746).
