# Supplementary material for: Impact of additives on syntrophic propionate and acetate enrichments under high-ammonia conditions
Source: Appl Microbiol Biotechnol. 2024 Aug 7;108:433. doi: 10.1007/s00253-024-13263-7 (PMC11306274; doi:10.1007/s00253-024-13263-7)
Supplement: Supplementary file 1 [file 253_2024_13263_MOESM1_ESM.pdf]

Supplementary Information (SI)

Applied Microbiology and Biotechnology

Impact of additives on syntrophic propionate and acetate enrichments under high-ammonia conditions

Eduardo Pinela[1], Anna Schnürer[1], Anna Neubeck[2], Jan Moestedt[1,3,4], Maria Westerholm[1, *]

[1]Department of Molecular Sciences, Swedish University of Agricultural Sciences, Uppsala, SE-750 07, Sweden

[2]Department of Earth Sciences, Uppsala University, Uppsala SE-752 36, Sweden

[3]Department of Biogas R & D, Tekniska verken i Linköping AB (publ.), Box 1500, Linköping, SE-581 15, Sweden

[4]Department of Thematic Studies - Environmental Change, Linköping University, Linköping SE-581 83, Sweden

***Correspondence:**
Maria Westerholm
Maria.Westerholm@slu.se ORCID: 0000-0003-2150-8762

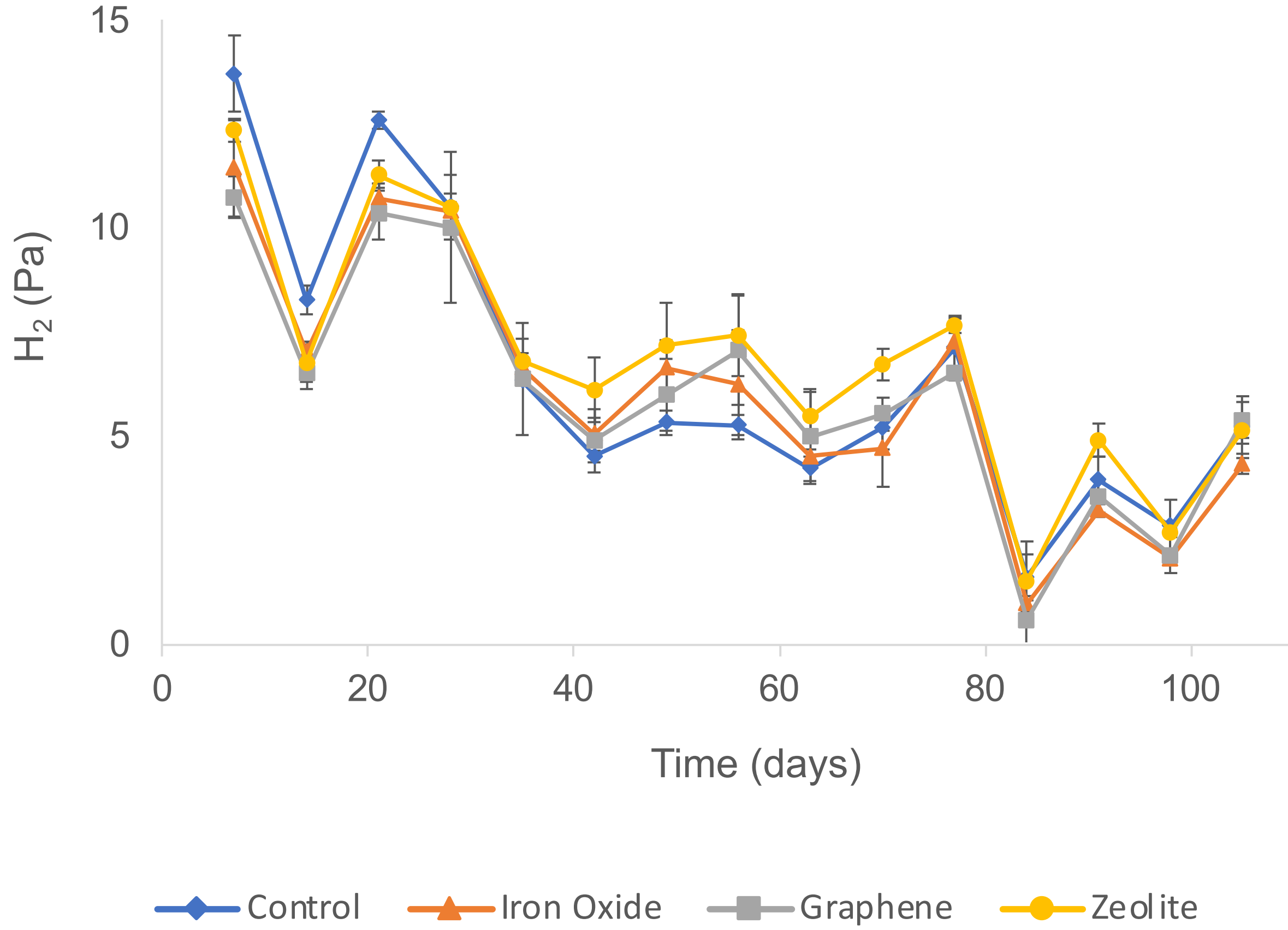

Fig. S1 Variation in H_2 partial pressure (Pa) during the first round of acetate degradation (A1) in syntrophic acetate-oxidising (SAO) enrichment cultures

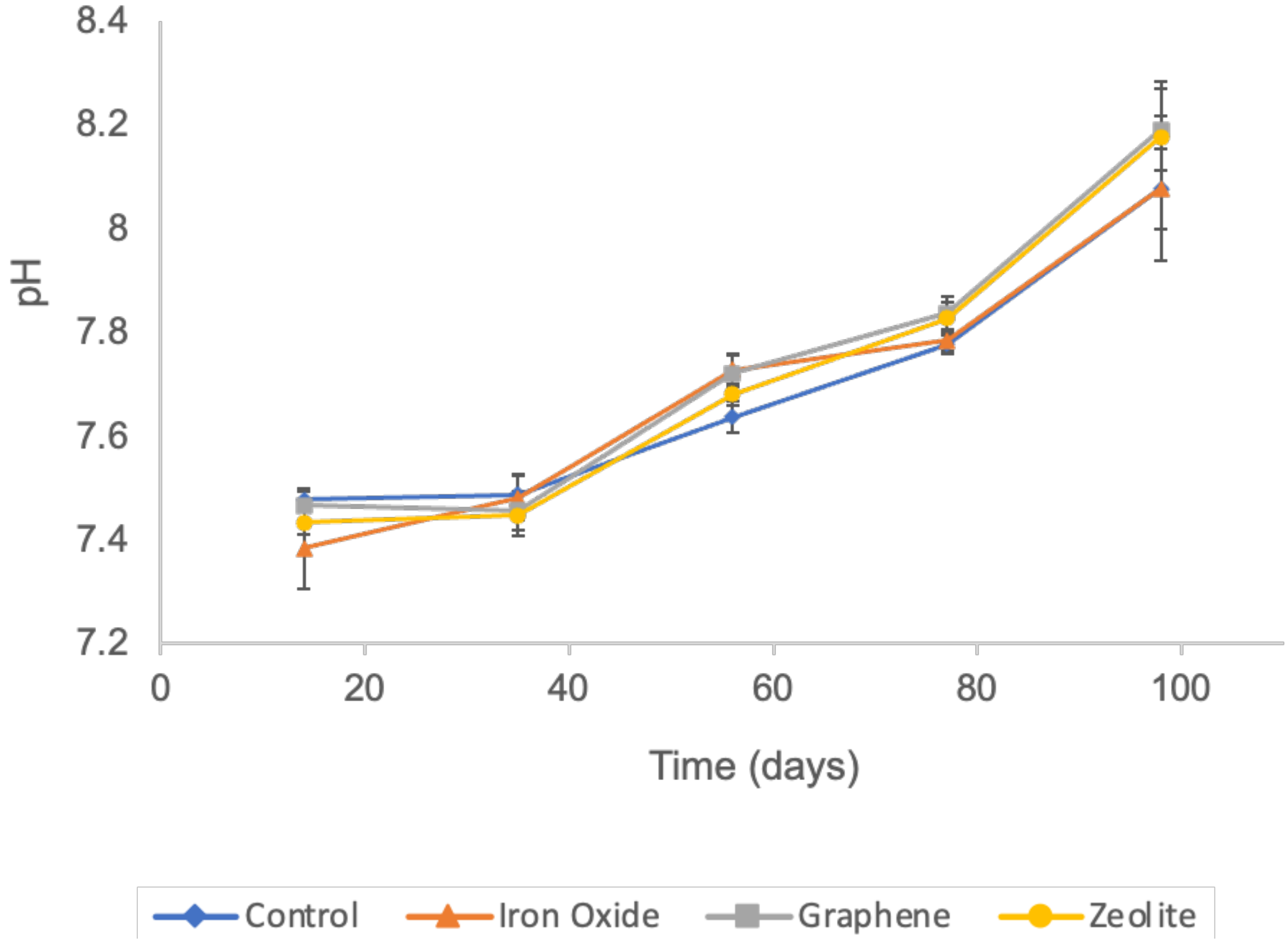

Fig. S2 Variation in pH during the first round of acetate degradation (A1) in syntrophic acetate-oxidising (SAO) enrichment cultures

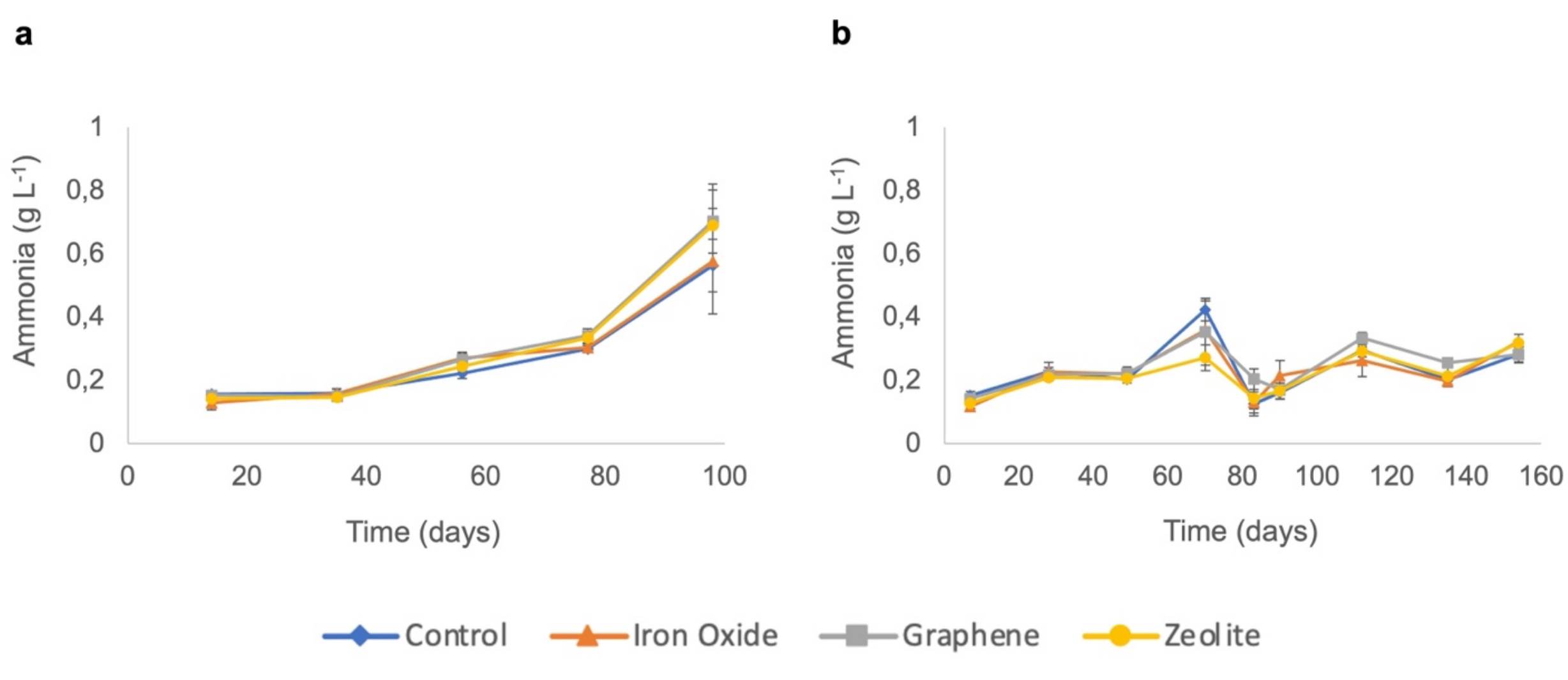

Fig. S3 Variation in ammonia concentration during the first round of (**a**) acetate degradation (A1) in syntrophic acetate-oxidising (SAO) enrichment cultures and (**b**) propionate degradation (P1) in syntrophic propionate-oxidising (SPO) enrichment cultures. Values calculated by entering pH values into the equation in Table S4

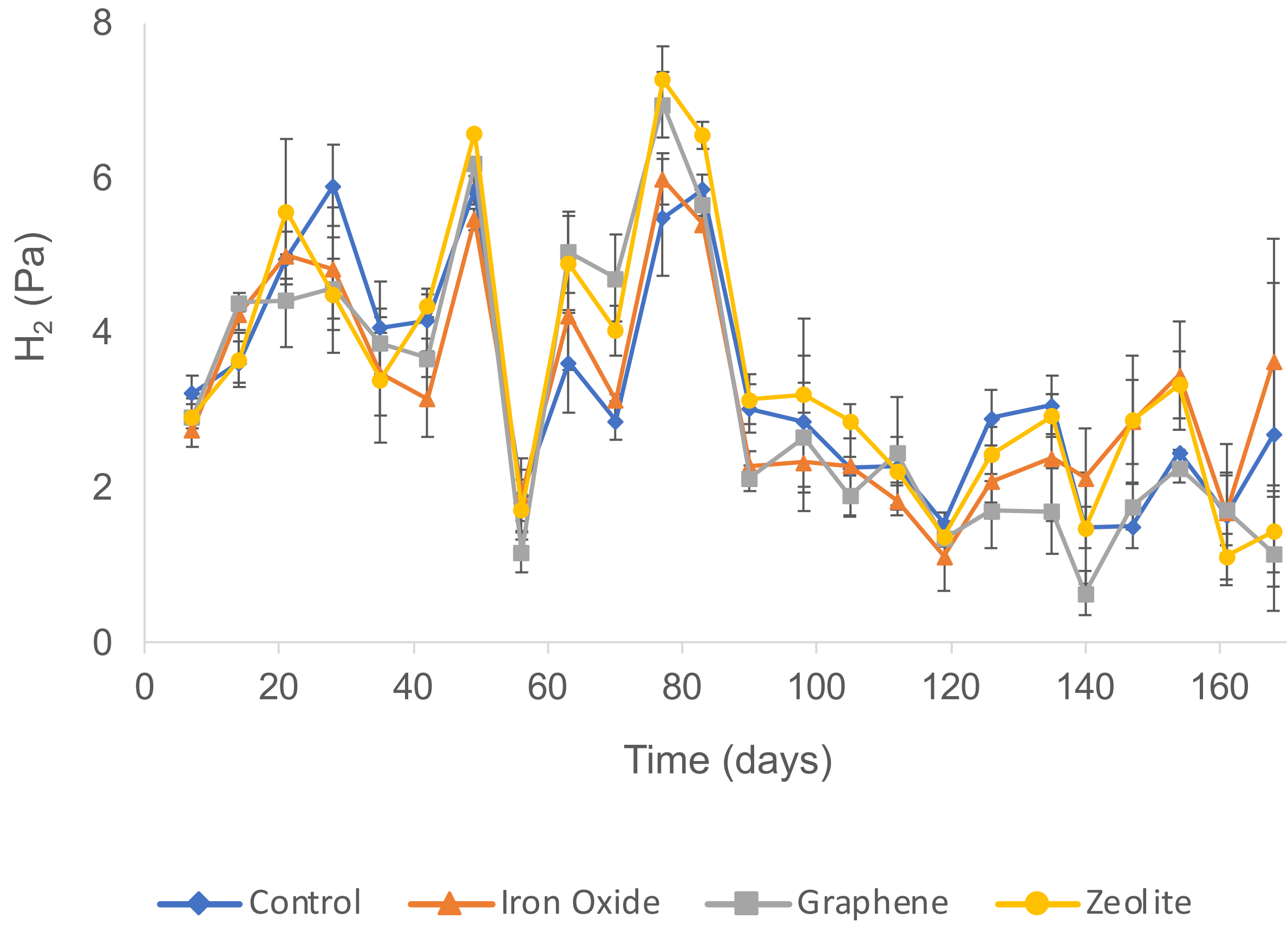

Fig. S4 Variation in H_2 partial pressure (Pa) during the first round of propionate degradation (P1) in syntrophic propionate-oxidising (SPO) enrichment cultures

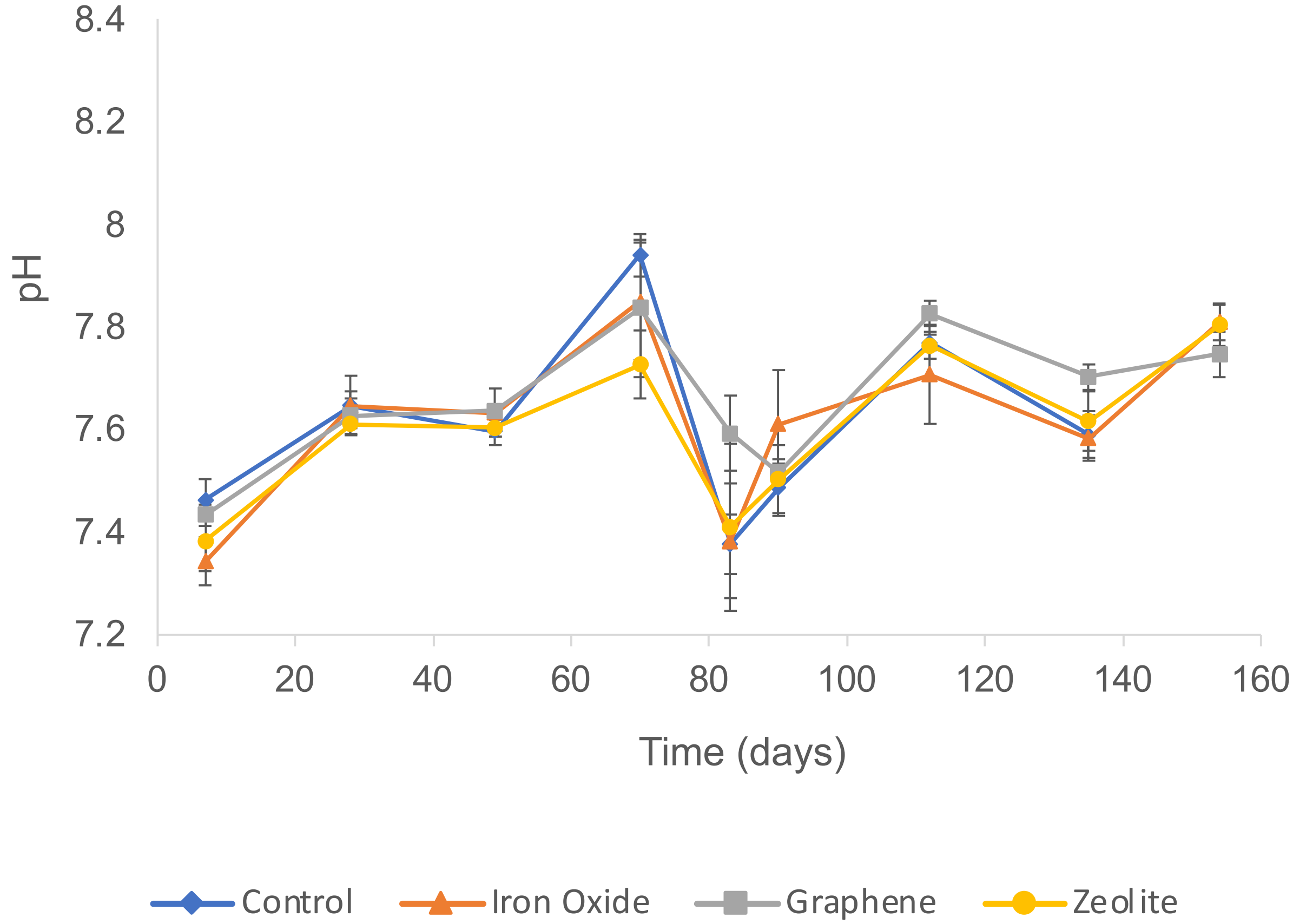

Fig. S5 Variation in pH during the first round of propionate degradation (P1) in syntrophic propionate-oxidising (SPO) enrichment cultures

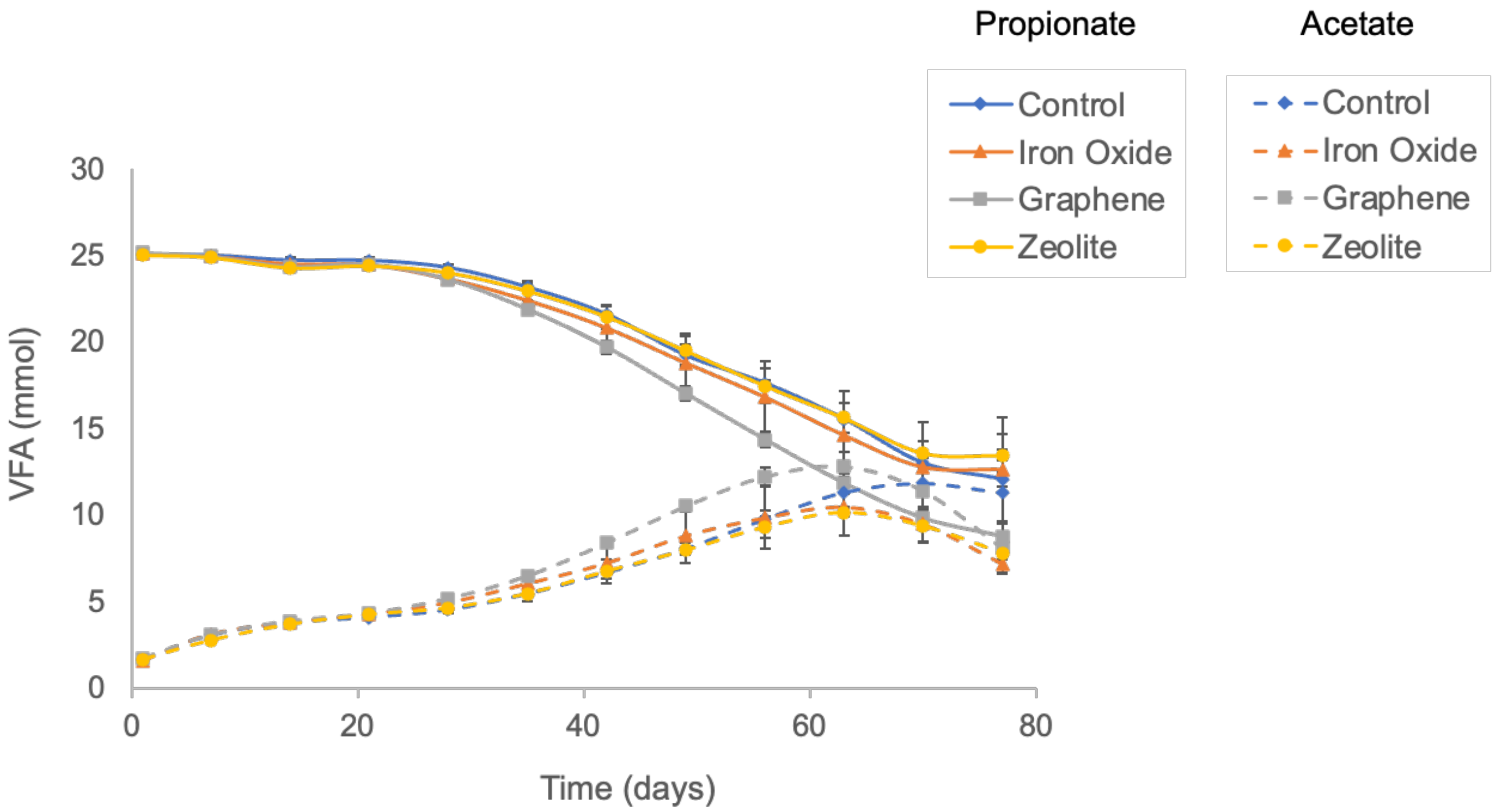

Fig. S6 Molar conversion of propionate into acetate up to peak acetate level (between days 63-70) during the first round of propionate degradation (P1) in syntrophic propionate-oxidising (SPO) enrichment cultures

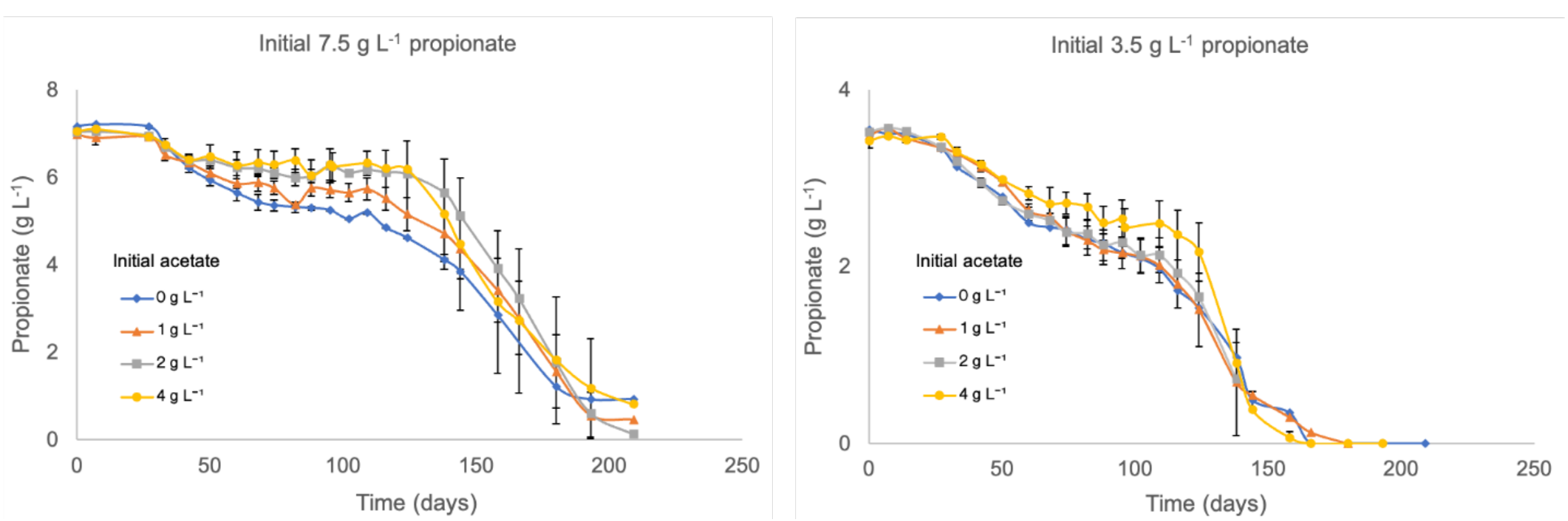

Fig. S7 Impact of acetate (initial concentration 0, 1, 2 or 4 g L^{-1}) on propionate degradation (initial concentration of 7.5 or 3.5 g L^{-1}). Note the different scale on the y-axes

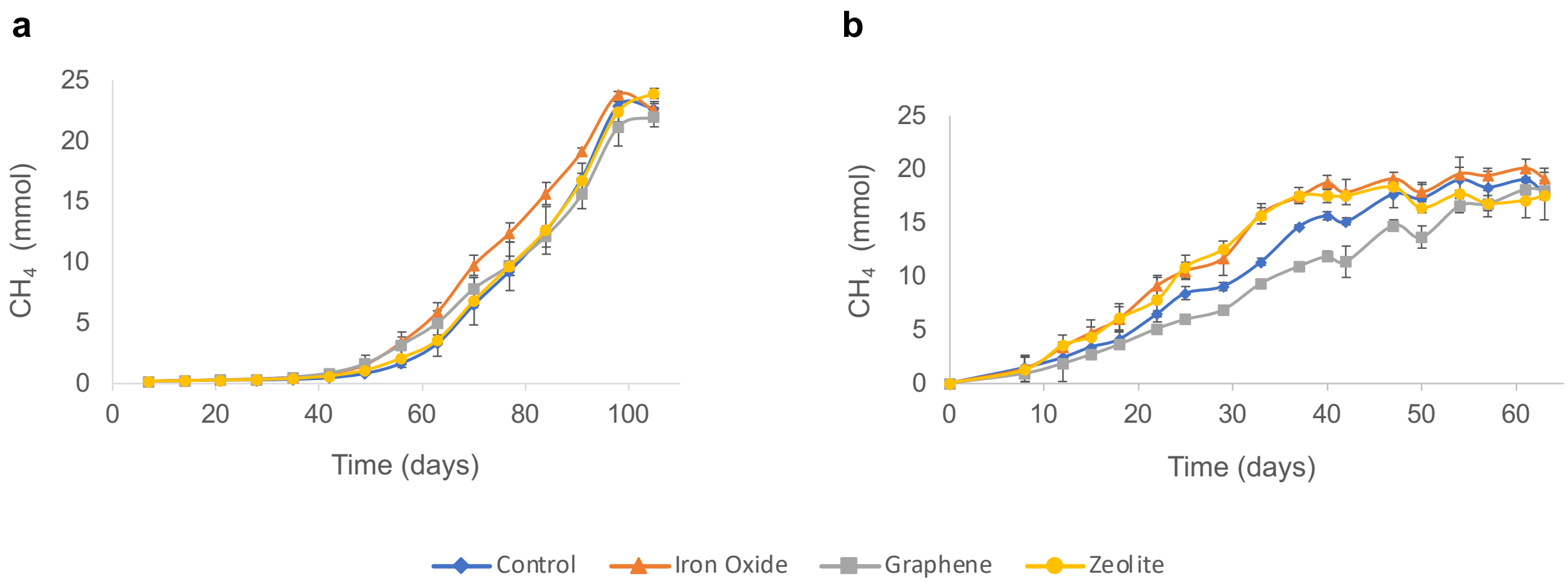

Fig. S8 Moles of methane produced in the acetate-fed batches during (**a**) the first (A1) and (**b**) the second (A2) round of acetate degradation

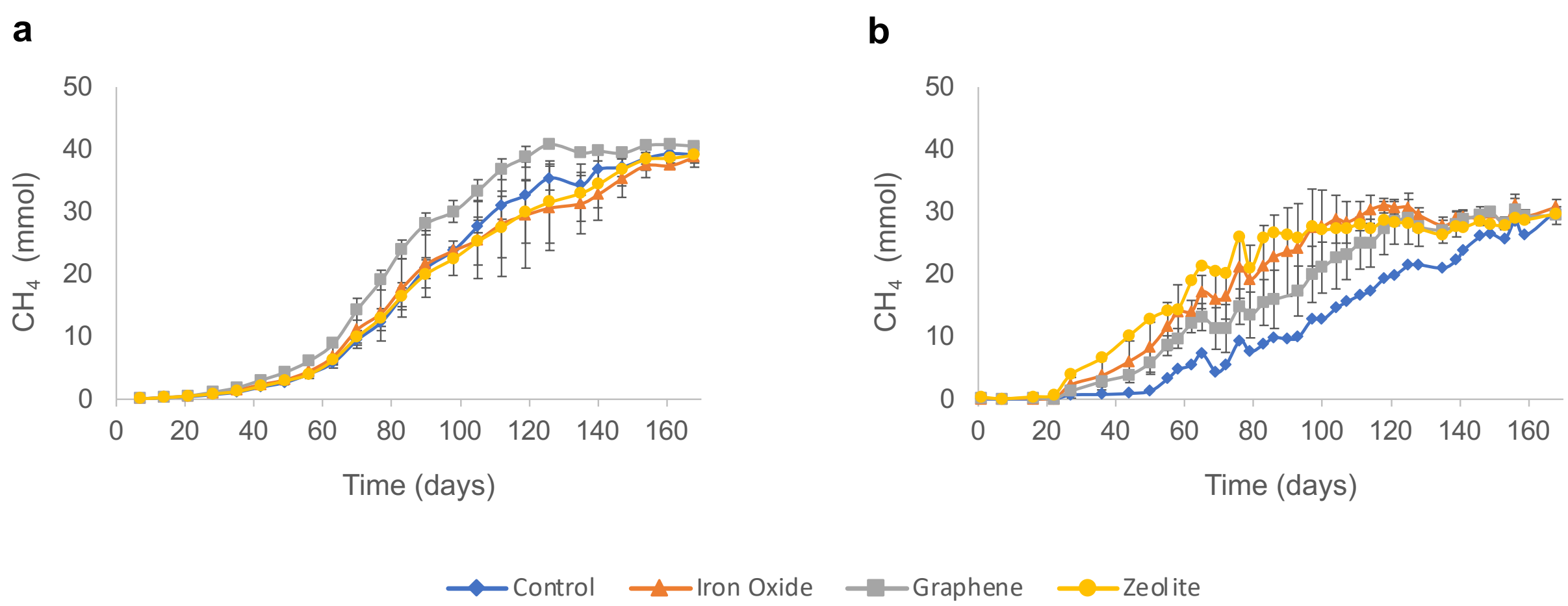

Fig. S9 Moles of methane produced in the propionate-fed batches during (**a**) the first (P1) and (**b**) the second (P2) round of propionate degradation. Averages for the control and zeolite batches during P2 were calculated using duplicates

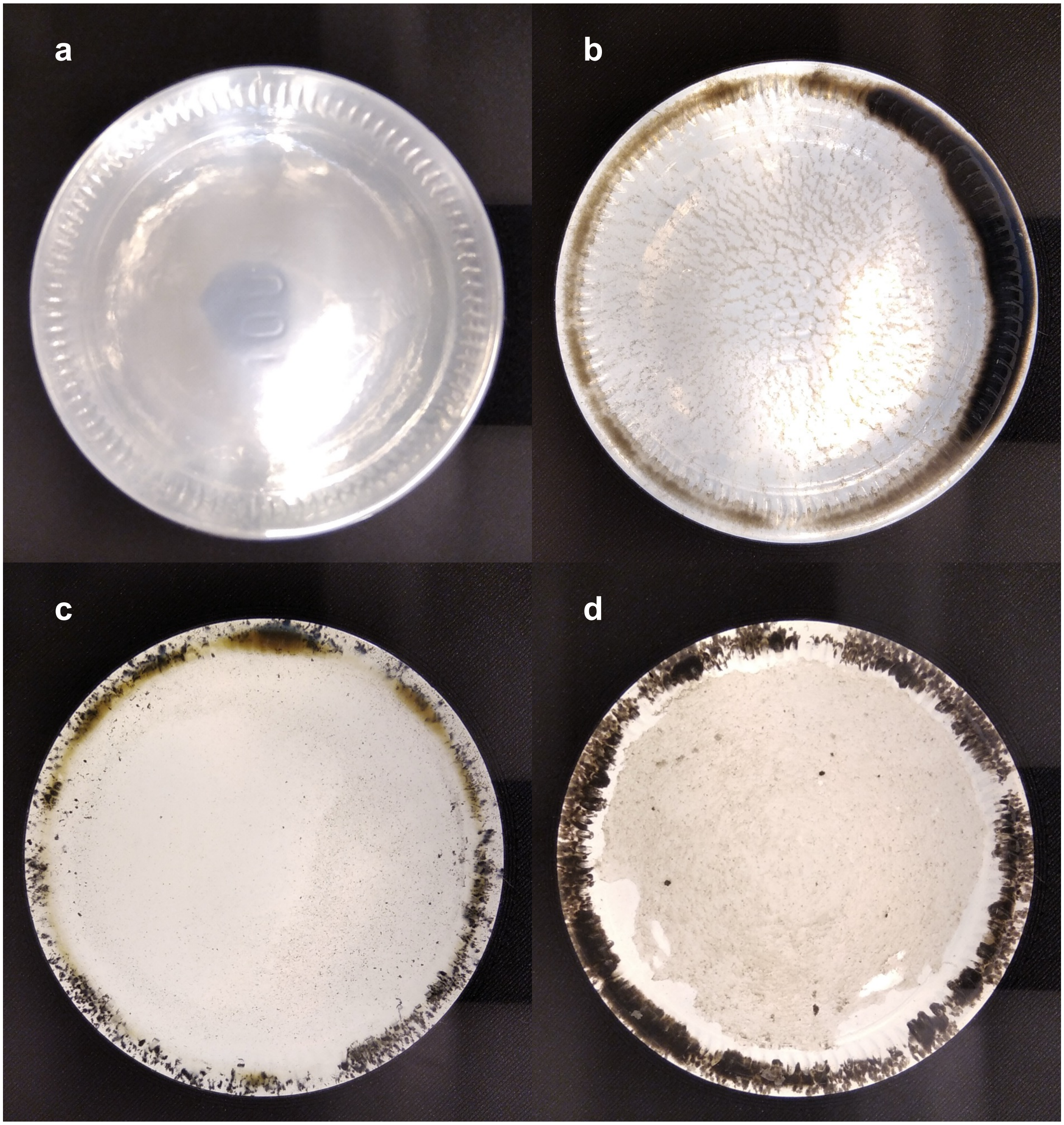

Fig. S10 Biomass observable to the naked eye at the start of the first round of (**a**) acetate (A1) and (**b**) propionate (P1) degradation, and at the end of the second round of (**c**) acetate (A2) and (**d**) propionate (P2) degradation in the syntrophic acetate-oxidising (SAO) and syntrophic propionate-oxidising (SPO) enrichment cultures, respectively. Images (**a**) and (**b**) were obtained at day 1 and day 3 of A1 and P1, respectively, while images (**c**) and (**d**) were obtained at day 104 and day 168 of A2 and P2, respectively

Table S1 Experimental set-up of batch trials investigating the impact of presence of acetate without addition of supportive materials on propionate degradation. All batch sets were run in triplicate

Batch set	Propionate mM (g L^{-1})	Acetate mM (g L^{-1})
1	47 (3.5)	0
2	47 (3.5)	14 (0.8)
3	47 (3.5)	29 (1.7)
4	47 (3.5)	61 (3.6)
5	95 (7.1)	0
6	95 (7.1)	14 (0.8)
7	95 (7.1)	29 (1.7)
8	95 (7.1)	61 (3.6)

Table S2 Values used in calculation of acid degradation rates during the first (P1) and second (P2) rounds of propionate degradation and during the first (A1) and second (A2) rounds of acetate degradation

Round	Acid	Batch set	Acid level at first day of linear degradation phase (g L^{-1})	Acid level at last day of linear degradation phase (g L^{-1})	Number of days in between two acid levels	Rate of degradation (g L^{-1} day^{-1})
First	Propionate	Control 1	3.279	1.821	35	0.042
		Control 2	3.255	1.892	35	0.039
		Control 3	3.219	1.815	35	0.040
		Iron Oxide 1	3.049	1.389	35	0.047
		Iron Oxide 2	3.146	2.061	35	0.031
		Iron Oxide 3	3.248	1.984	35	0.036
		Graphene 1	3.073	1.348	35	0.049
		Graphene 2	3.045	1.443	35	0.046
		Graphene 3	3.107	1.385	35	0.049
		Zeolite 1	3.281	2.027	35	0.036
		Zeolite 2	3.253	1.903	35	0.039
		Zeolite 3	3.139	1.845	35	0.037
Second	Propionate	Control 1	3.265	1.914	14	0.096
		Control 2	3.158	1.834	14	0.095
		Control 3	N/A	N/A	N/A	N/A
		Iron Oxide 1	1.803	0.134	14	0.119
		Iron Oxide 2	3.164	1.409	14	0.125

		Iron Oxide 3	2.760	0.739	14	0.144
		Graphene 1	2.906	1.599	14	0.093
		Graphene 2	3.165	1.781	14	0.099
		Graphene 3	2.633	1.118	14	0.108
		Zeolite 1	N/A	N/A	N/A	N/A
		Zeolite 2	1.524	0.200	14	0.095
		Zeolite 3	1.865	0.157	14	0.122
First	Acetate	Control 1	2.484	0.744	21	0.083
		Control 2	2.502	0.748	21	0.084
		Control 3	2.544	0.811	21	0.083
		Iron Oxide 1	2.115	0.333	21	0.085
		Iron Oxide 2	1.997	0.252	21	0.083
		Iron Oxide 3	2.179	0.447	21	0.082
		Graphene 1	2.419	0.748	21	0.080
		Graphene 2	2.062	0.727	21	0.064
		Graphene 3	2.282	1.094	21	0.057
		Zeolite 1	2.638	1.032	21	0.077
		Zeolite 2	2.213	0.396	21	0.087
		Zeolite 3	2.529	0.827	21	0.081
Second	Acetate	Control 1	2.847	0.924	25	0.077
		Control 2	2.891	0.890	25	0.080
		Control 3	3.181	1.002	25	0.087
		Iron Oxide 1	2.798	0.338	25	0.098
		Iron Oxide 2	2.799	0.455	25	0.094
		Iron Oxide 3	2.878	0.330	25	0.102
		Graphene 1	2.899	1.433	25	0.059
		Graphene 2	2.540	1.209	25	0.053
		Graphene 3	3.271	1.420	25	0.074
		Zeolite 1	2.697	0.361	25	0.093
		Zeolite 2	3.238	0.198	25	0.122
		Zeolite 3	2.850	0.100	25	0.110

Table S3 Carbon balance calculations during the first (P1) and second (P2) rounds of propionate degradation and during the first (A1) and second (A2) rounds of acetate degradation. Theoretical moles of CH_4 were calculated using conversion coefficients of 1.75 and 1 from propionate and acetate, respectively

Round	Acid	Batch set	Starting moles of acid (mmol)	Theoretical moles of CH4 (mmol)	Moles of CH4 obtained (mmol)	Yield (%)
First	Propionate	Control 1	25	43.75	37.56	85.85
		Control 2	25	43.75	39.04	89.23
		Control 3	25	43.75	40.81	93.27
		Iron Oxide 1	25	43.75	39.63	90.59
		Iron Oxide 2	25	43.75	38.14	87.18
		Iron Oxide 3	25	43.75	38.67	88.39
		Graphene 1	25	43.75	40.90	93.49
		Graphene 2	25	43.75	40.25	92.01
		Graphene 3	25	43.75	40.57	92.74
		Zeolite 1	25	43.75	39.89	91.19
		Zeolite 2	25	43.75	41.03	93.78
		Zeolite 3	25	43.75	36.49	83.40
Second	Propionate	Control 1	25	43.75	34.83	79.61
		Control 2	25	43.75	28.65	65.47
		Control 3	25	43.75	27.79	63.53
		Iron Oxide 1	25	43.75	32.09	73.35
		Iron Oxide 2	25	43.75	29.69	67.87
		Iron Oxide 3	25	43.75	30.86	70.55
		Graphene 1	25	43.75	30.96	70.76
		Graphene 2	25	43.75	29.68	67.84
		Graphene 3	25	43.75	28.10	64.24
		Zeolite 1	25	43.75	24.96	57.06
		Zeolite 2	25	43.75	33.09	75.64
		Zeolite 3	25	43.75	31.51	72.02
First	Acetate	Control 1	25	25	22.53	90.12
		Control 2	25	25	23.26	93.06
		Control 3	25	25	22.14	88.55
		Iron Oxide 1	25	25	22.55	90.18
		Iron Oxide 2	25	25	23.37	93.50

		Iron Oxide 3	25	25	21.70	86.81
		Graphene 1	25	25	23.04	92.16
		Graphene 2	25	25	21.54	86.16
		Graphene 3	25	25	21.27	85.09
		Zeolite 1	25	25	24.31	97.23
		Zeolite 2	25	25	23.40	93.59
		Zeolite 3	25	25	24.04	96.14
Second	Acetate	Control 1	25	25	19.44	77.77
		Control 2	25	25	17.76	71.03
		Control 3	25	25	19.81	79.25
		Iron Oxide 1	25	25	19.42	77.70
		Iron Oxide 2	25	25	19.83	79.31
		Iron Oxide 3	25	25	21.02	84.06
		Graphene 1	25	25	18.17	72.68
		Graphene 2	25	25	18.49	73.95
		Graphene 3	25	25	17.77	71.07
		Zeolite 1	25	25	15.33	61.32
		Zeolite 2	25	25	18.66	74.65
		Zeolite 3	25	25	17.30	69.19

Table S4 Equation and variables used in calculation of ammonia values

Temperature (K)	273.15 + Temperature (°C)
pKa	$0.09018 + \left(\frac{2729.92}{\text{Temperature (K)}}\right)$
NH_3 (g L^{-1})	$\frac{NH_4\ (\text{g } L^{-1})}{\left(1+10^{(pKa-pH)}\right)}$
